# Pathogenic myelin-specific antibodies in multiple sclerosis target conformational proteolipid protein 1–anchored membrane domains

**DOI:** 10.1172/JCI162731

**Published:** 2023-10-02

**Authors:** Gregory P. Owens, Timothy J. Fellin, Adeline Matschulat, Vanessa Salas, Kristin L. Schaller, Katherine S. Given, Alanna M. Ritchie, Andre Navarro, Kevin Blauth, Ethan G. Hughes, Wendy B. Macklin, Jeffrey L. Bennett

**Affiliations:** 1 Department of Neurology,; 2 Department of Cell & Developmental Biology,; 3 Program in Neuroscience,; 4 Department of Ophthalmology, and; 5 Program in Immunology, University of Colorado School of Medicine, Anschutz Medical Campus, Aurora, Colorado, USA.

**Keywords:** Autoimmunity, Neuroscience, Antigen, Immunoglobulins, Multiple sclerosis

## Abstract

B cell clonal expansion and cerebrospinal fluid (CSF) oligoclonal IgG bands are established features of the immune response in multiple sclerosis (MS). Clone-specific recombinant monoclonal IgG1 Abs (rAbs) derived from MS patient CSF plasmablasts bound to conformational proteolipid protein 1 (PLP1) membrane complexes and, when injected into mouse brain with human complement, recapitulated histologic features of MS pathology: oligodendrocyte cell loss, complement deposition, and CD68^+^ phagocyte infiltration. Conformational PLP1 membrane epitopes were complex and governed by the local cholesterol and glycolipid microenvironment. Abs against conformational PLP1 membrane complexes targeted multiple surface epitopes, were enriched within the CSF compartment, and were detected in most MS patients, but not in inflammatory and noninflammatory neurologic controls. CSF PLP1 complex Abs provide a pathogenic autoantibody biomarker specific for MS.

## Graphical abstract

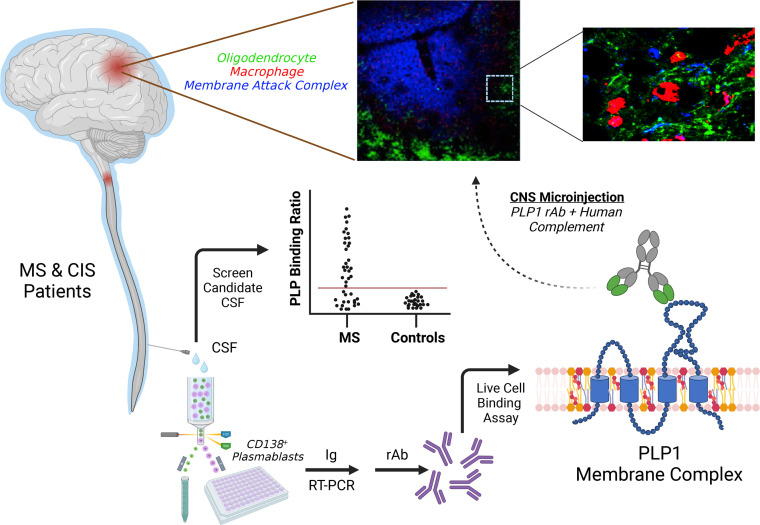

## Introduction

Multiple sclerosis (MS) is a chronic autoimmune inflammatory demyelinating disease of the CNS. Multiple molecular mechanisms have been posited as driving lesion pathology (1–3), but the failure to identify relevant CNS immune targets has significantly hampered a comprehensive understanding of disease pathogenesis and the development of targeted therapies.

One of the earliest biochemical observations of MS was the presence of cerebrospinal fluid (CSF) oligoclonal IgG bands (4). Oligoclonal bands (OCBs) are accompanied by elevated numbers of clonally expanded B cells and plasmablasts in CSF, meninges, and brain tissue (5–10). Deep sequencing of B cell repertoires established clonal relationships between CSF IgG OCBs and CSF B cell clones (11) and among CSF and meningeal and MS lesion B cell infiltrates (12). To better understand the intrathecal B cell response in MS, we constructed recombinant monoclonal IgG1 Abs (rAbs) from expanded CSF plasmablast clones (13) and demonstrated their differential patterns of binding to astrocytes, neurons, and myelin-enriched antigens (14). Although myelin-specific MS rAbs represent a smaller subset of observed specificities, they caused rapid oligodendrocyte cell death and myelin loss when applied to mouse spinal cord or cerebellar explant cultures in the presence of human complement (HC) (14, 15). Complement-mediated demyelination induced by myelin-specific MS Abs could promote the deposition of IgG and terminal complement products found in approximately 50% of active MS lesions (1), implying that intrathecal IgGs may play a direct role in CNS injury.

In this study, we demonstrate that myelin-specific MS rAbs target conformational membrane complexes containing the myelin proteolipid protein 1 (PLP1) and, in the presence of HC, are sufficient to drive in vivo demyelination. Recognition of PLP1 was strongly influenced by the lipid microenvironment. PLP1 complex–reactive Abs were also specific to MS, concentrated in the CSF, and present at multiple clinical stages of disease.

## Results

### Myelin-specific MS Abs mediate oligodendrocyte death in vivo.

**Figure 1:**
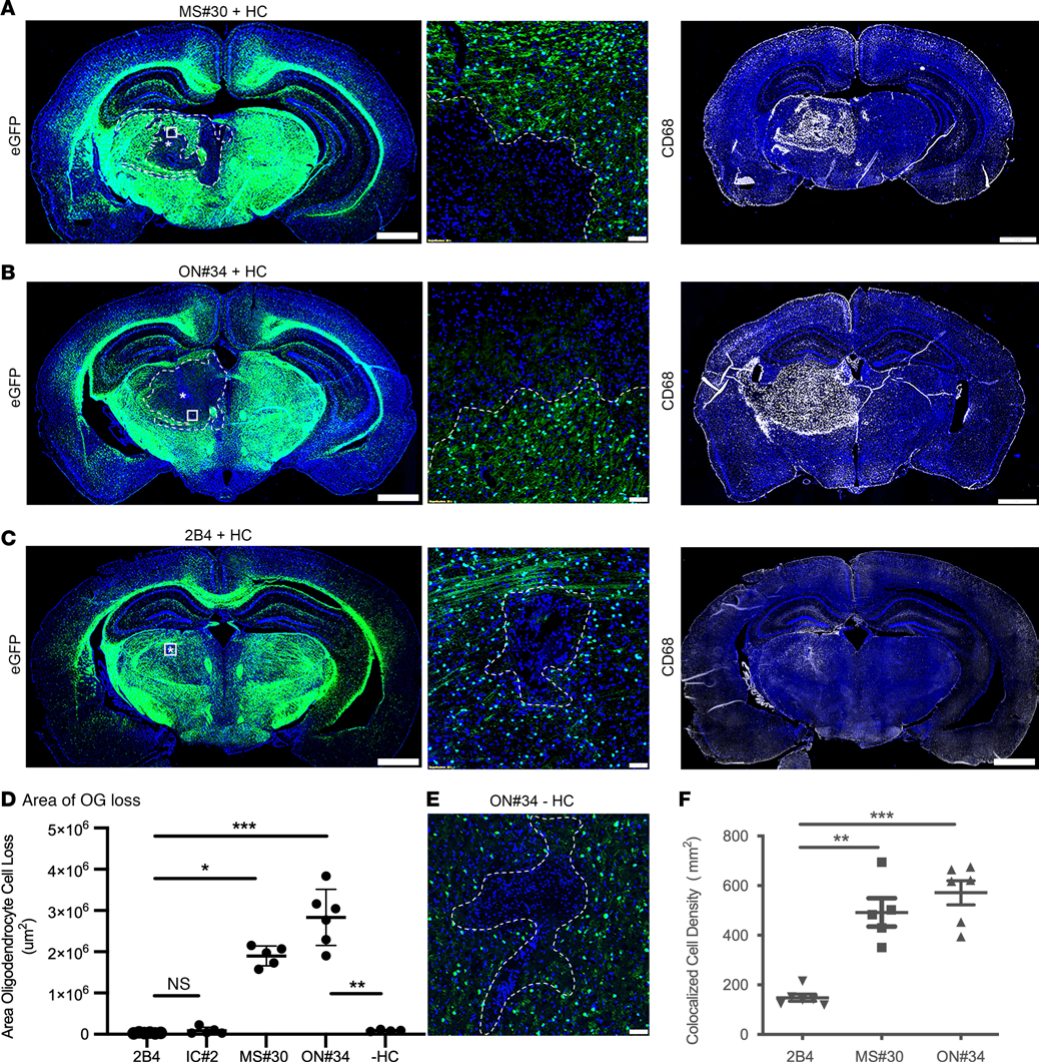
Myelin-specific rAbs initiate complement-dependent oligodendrocyte cell death. (**A**–**C**) EGFP immunofluorescence in brain sections of C57BL/6 PLP-EGFP mice following ICI of myelin-specific (MS#30, ON#34) or IC (2B4) rAbs with HC. (Left panels) Amorphous regions of EGFP^+^ oligodendrocyte loss at 72 hours after injection are demarcated by the dotted lines. Asterisks indicate injection site. (Center panels) Higher magnification images of boxed areas reveal a sharp demarcation between areas of complete oligodendrocyte loss and adjacent normal-appearing tissue. (Right panels) CD68^+^ microglia/macrophages accumulate within the lesion core. Scale bars: 1 mm (left and right); 50 µm (center). (**D**) Quantitation of the area of EGFP^+^ oligodendrocyte cell loss (4–6 animals per injection) for IC (2B4, IC#2) rAbs plus HC, myelin-specific (MS#30, ON#34) rAbs plus HC, and myelin-specific ON#34 rAb minus HC (–HC) (Kruskal-Wallis 1-way ANOVA with Dunn’s correction for multiple comparisons, *P < 0.05; ***P < 0.001; ON#34 +/– HC, Mann-Whitney *U* test, **P < 0.01). (**E**) High-magnification image of ON#34 rAb without HC (ON#34 – HC) ICI shows a minor loss of EGFP^+^ oligodendrocyte cell loss at the injection site. Scale bar: 50 µm. (**F**) Quantitation of CD68^+^DAPI^+^ cell density (per subcortical hemisphere) at 72 hours after injection following ICI of rAbs 2B4, MS#30, or ON#34, plus HC (ANOVA with Dunn’s correction for multiple comparisons, **P < 0.01).

Monoclonal rAbs targeting myelin-enriched antigens were previously identified from CSF plasmablast clones in 2 relapsing MS patients (Supplemental Table 1; supplemental material available online with this article; https://doi.org/10.1172/JCI162731DS1) (14, 15). MS rAb MS#30 was derived from patient MS04-2 and rAbs ON#34 and ON#49 were derived from patient ON07-7. To investigate their pathogenicity, we evaluated antibody-mediated CNS pathology in vivo following intracerebral injection (ICI) of IgG1 myelin–specific MS rAbs plus HC into the thalamus of C57BL/6 PLP-EGFP mice (16). Three days after injection of myelin-specific rAbs MS#30 and ON#34, there was nearly complete loss of EGFP^+^ oligodendrocytes surrounding the injection site (Figure 1, A, B, and D); minimal oligodendrocyte loss was observed with isotype-control (IC) rAbs 2B4 and IC#2 (Figure 1, C and D). Similar oligodendrocyte cell loss was observed following ICI of ON#49+HC (not shown). Demyelinating lesions showed infiltrating CD68^+^ phagocytes (Figure 1, A, B, and F) that were accentuated along the lesion edge and actively engaged in myelin and oligodendrocyte phagocytosis in areas of IgG and complement deposition (Supplemental Figure 1). Lesion formation was dependent on HC and not observed following injection of ON#34 alone (Figure 1, D and E).

Immunostaining with astrocytic markers aquaporin-4 (AQP4) and glial fibrillary acidic protein (GFAP) revealed an absence of staining within areas of oligodendrocyte cell loss and accentuated GFAP staining adjacent to the lesion edge (Supplemental Figure 2A), suggesting an early coincidental loss of astrocytes in the lesion core and astrogliosis along the lesion periphery. To confirm this loss of astrocytes, we repeated MS#30 ICIs in Tg(Aldh1l1-EGFP,-DTA)D8Rth Swiss Webster mice. Animals were evaluated for areas of EGFP^+^ astrocyte loss at 3, 7, and 14 days after injection; day 3 2B4 rAb ICIs were evaluated as controls. CD68 cell infiltration and astrocyte loss were minimal and associated exclusively with the injection tract in 2B4 day-3 ICIs. Significant loss of EGFP^+^ astrocyte cell bodies relative to 2B4 controls was observed within areas of CD68 cell infiltration in day-3 MS#30 lesions (Supplemental Figure 2, B–D). Patchy return of EGFP^+^ astrocytes into areas of CD68^+^ cell infiltration was observed on day 7 and was more pronounced in areas distant from the lesion center (Supplemental Figure 2, B–D). Astrocyte recovery was near complete (>90%) by day 14 (Supplemental Figure 2, C and D), and the only notable region of astrocyte loss remained in the permanently damaged injection site. At day 7, astrocytes infiltrating the lesion site displayed an activated phenotype with enhanced GFAP expression relative to surrounding CNS tissue. This phenotype of active astrocyte gliosis persisted within day-14 lesions (Supplemental Figure 2D).

### Myelin-specific rAbs bind to the cell surface of PLP1-expressing cells.

**Figure 2:**
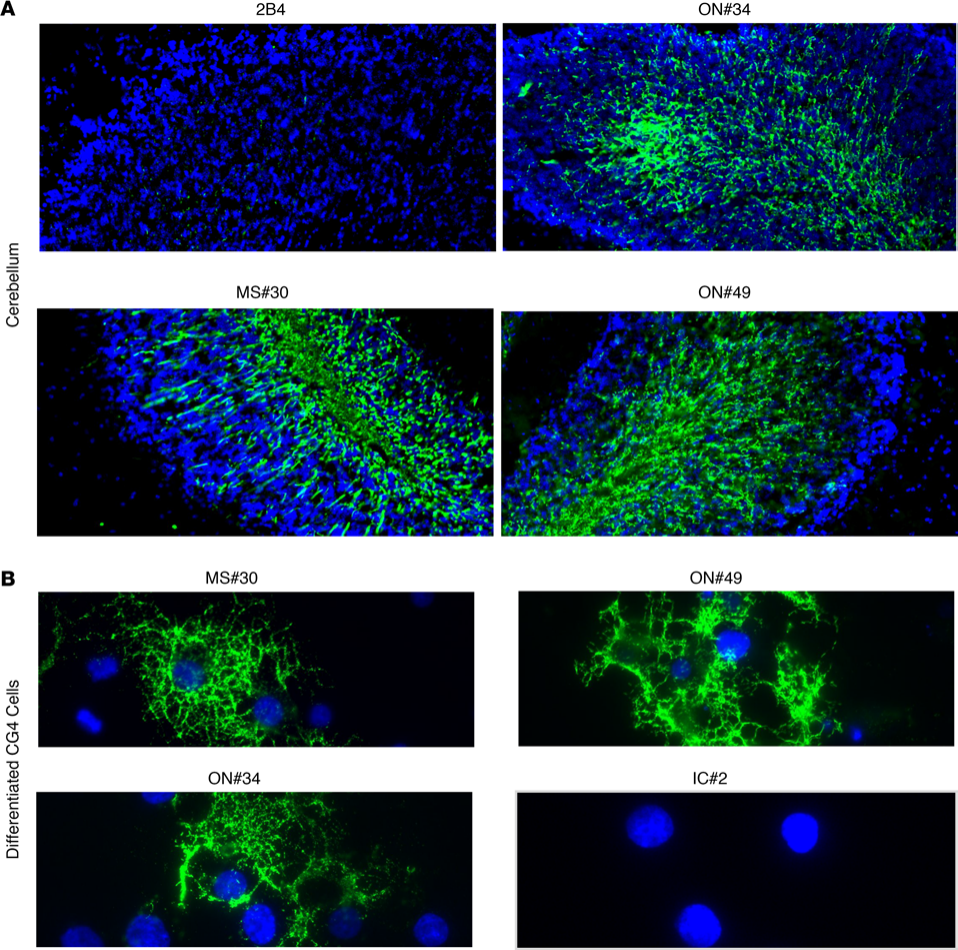
MS rAbs bind to myelinated axons and the surface of live differentiated CG4 oligodendrocytes. (**A**) MS (MS#30, ON#49, and ON#34) and IC (2B4) rAb (20 μg/ml) immunofluorescence in murine cerebellum. Original magnification, ×200. (**B**) MS rAbs MS#30, ON#49, and ON#34 and control IC#2 rAb immunofluorescence on live CG4 rat oligodendrocyte cultures 48 hours after differentiation. Original magnification, ×400. All sections are counterstained with DAPI.

**Figure 3:**
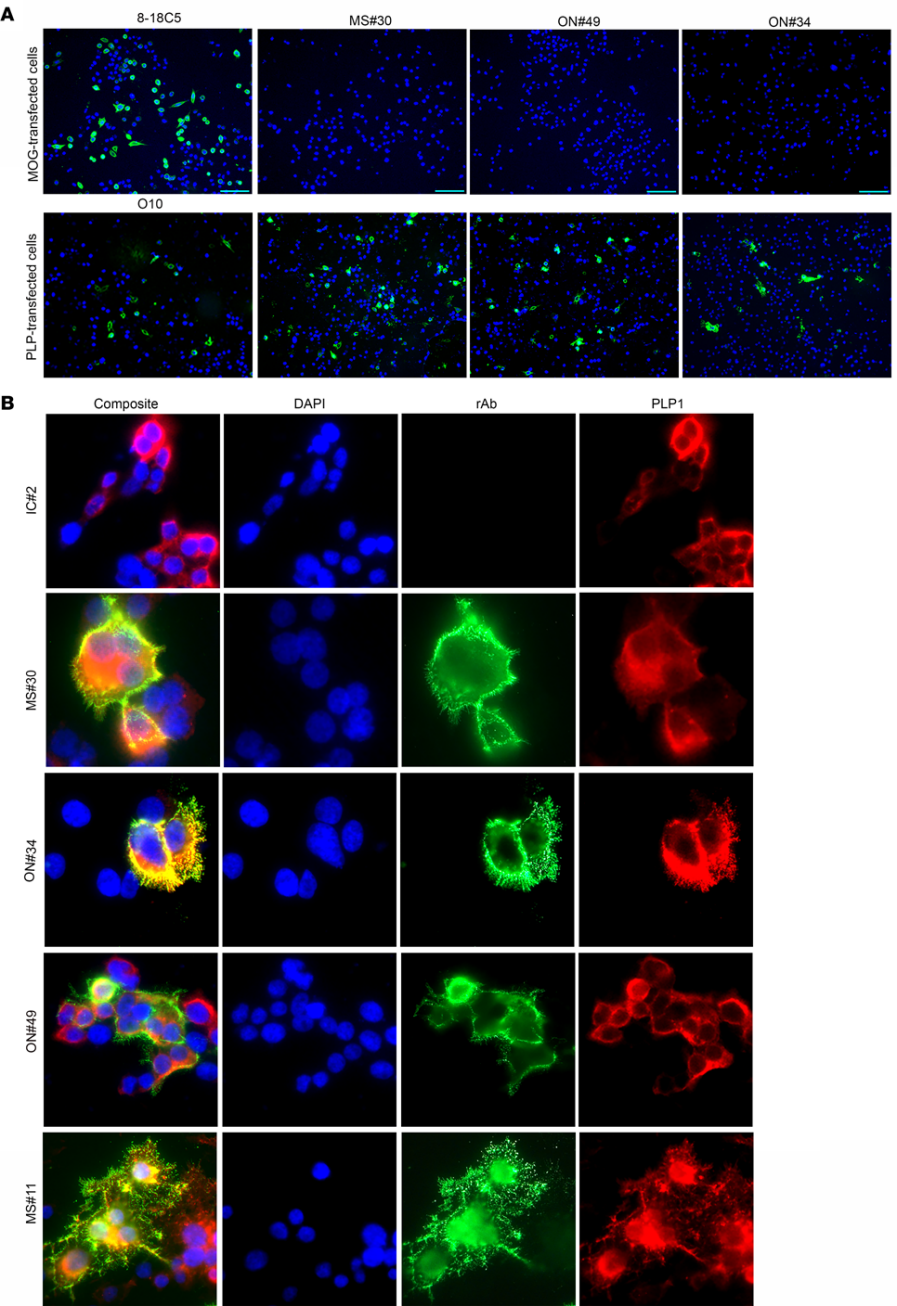
MS rAbs bind to the surface of PLP1-transfected cells. (**A**) Live-cell binding of myelin-specific rAbs (MS#30, ON#49, and ON#34) to CHOK1 cells transfected with MOG or PLP1 expression vector plasmid DNA at 24 hours after transfection. Live-cell staining with MOG-specific 8-18C5 mAbs (10 µg/ml) or PLP1-specific O10 mAbs (1:2 dilution of hybridoma supernatant) serve as positive controls. Scale bars: 25 µm. (**B**) Live-cell immunofluorescence (×600) of IC and myelin-specific MS rAbs (MS#30, ON#49, ON#34, and MS#11) in HEKE cells expressing PLP1. Human rAb binding is shown in green (rAb) and staining with an intracellular epitope-specific PLP1 mAb (plp1c or AA3) is shown in red (PLP1).

Myelin-specific rAbs MS#30, ON#34, and ON#49 bound myelinated tracts in murine tissue and stained surface antigen in a subset of live differentiating rat CG4 oligodendrocytes (Figure 2). The ability of myelin-specific rAbs to bind the surface of myelinated axons and oligodendrocyte processes in cerebellar explants (15) and live CG4 cells suggested that the antigen target was located on the outer myelin membrane surface. Live-cell staining of HEK293E cells expressing the myelin proteins MOG or PLP1 showed surface binding of MS rAbs to PLP1, but not to MOG-transfected cells (Figure 3A). The specificity of each transfection was confirmed by live staining with MOG-specific (8-18C5) or PLP1-specific (O10) mAbs. MS rAb staining, but not IC IC#2, visibly colocalized with PLP1 expression following double labeling of cells with an intracellular C-terminal PLP1-specific mAb (Figure 3B). Rescreening our panel of CSF-derived rAbs identified an additional rAb, MS#11 (patient MS04-2, Supplemental Table 1), that bound to the surface of live PLP1-transfected cells (Figure 3B). Like rAbs MS#30, ON#34, and ON#49, MS#11 caused significant oligodendrocyte cell death, CD68^+^ cell infiltration, and loss of AQP4 astrocyte staining following ICI with HC (Supplemental Figure 3). Overall, 4 of 37 MS CSF–derived rAbs and 0 of 18 inflammatory control CSF-derived rAbs (neuromyelitis optica [NMO], subacute sclerosing panencephalitis [SSPE], chronic meningitis, pediatric opsoclonus myoclonus syndrome) visibly bound to PLP1-transfected cells.

### MS rAbs recognize conformational PLP1 cell-surface epitopes.

MS rAb binding to live PLP1-transfected cells was dependent on the expression of spatially complex, surface-membrane epitopes. MS rAb staining was lost following treatment with organic fixatives, including methanol and acetone, and was only partially retained following paraformaldehyde (PFA) fixation (Supplemental Figure 4). Among Chinese hamster ovary (CHOK1) cells transfected with PLP1, only a fraction (30%–60%) were surface stained by individual myelin-specific MS rAbs (Supplemental Figure 4C). We quantified the fraction of PLP1-transfected cell staining with MS rAbs in either live or permeabilized PFA-fixed cells to ascertain whether access to intracellular PLP1 protein improved MS rAb binding. PFA fixation did not improve binding of MS rAbs; instead, it reduced the fraction of positive cells, most notably for MS#11 (Supplemental Figure 4C).

### Myelin-specific rAbs do not bind CNS white matter or promote complement-mediated oligodendrocyte cell death in PLP1-null animals.

**Figure 4:**
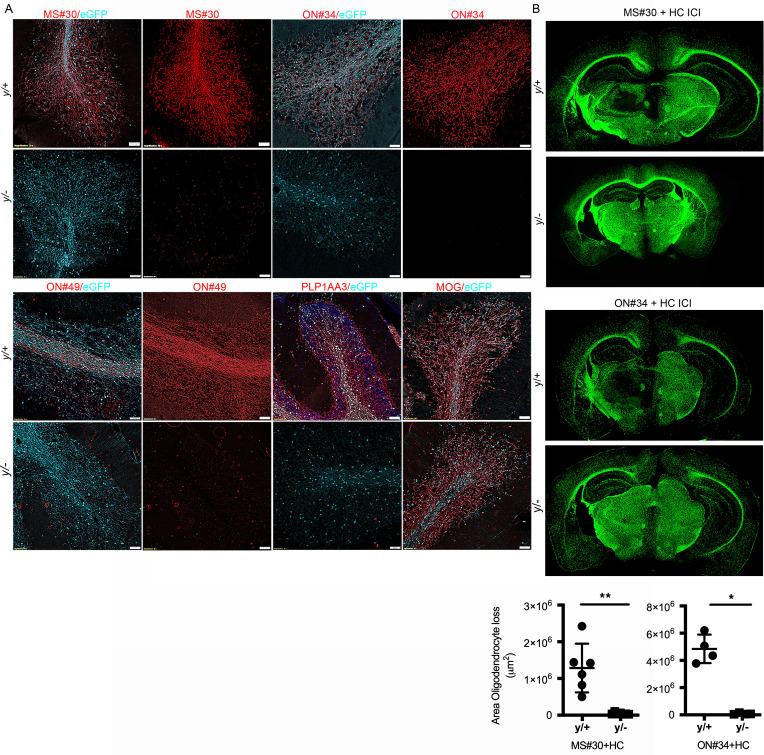
Myelin-specific MS rAbs do not bind myelin or mediate oligodendrocyte cell death in PLP1-null animals. (**A**) Cerebellar sections from WT (PLP^y/+^) and null (PLP^y/–^) male EGFP-PLP knockin mice were stained with myelin-specific MS rAbs (MS#30, ON#49, ON34), anti-PLP1 mAb AA3 (PLP1), and anti-MOG 8-18C5 mAbs. Scale bars: 50 µm. (**B**) EGFP^+^ immunofluorescence (×100) in murine brain following ICI of MS#30 or ON#34 rAbs with HC into male WT (PLP^y/+^) and hemizygous null (PLP^y/–^) EGFP-PLP knockin mice. Quantitation of the area of oligodendrocyte loss in PLP^y/+^ and PLP^y/–^ animals injected with MS#30 plus HC (*n* = 6 PLP^y/+^ and 7 PLP^y/^ ICIs) and ON#34 plus HC (*n* = 4 PLP^y/+^ and 4 PLP^y/–^ ICIs) (Mann-Whitney *U* test, *P < 0.05; **P < 0.01).

To confirm PLP1 as the primary target of these pathogenic demyelinating Abs, we stained cerebellar tissue from WT (PLP^y/+^) and hemizygous null (PLP^y/–^) male EGFP-PLP knockin mice (Figure 4A). Strong binding to white matter and EGFP^+^ myelinated Purkinje cell axons was observed with MS#30, ON#49, and ON#34 rAbs in PLP^y/+^ but not PLP^y/–^ animals. This same pattern of differential staining was also found with a positive control PLP mAb (AA3), whereas MOG-specific mAbs stained myelinated tracts in both PLP^y/+^ and PLP^y/–^ cerebelli. Due to its location on the X chromosome and X-linked inactivation, PLP expression is mosaic within the CNS of female PLP^+/–^ animals. This was reflected in the staining of PLP^+/–^ cerebellar slice cultures where MS#30 staining colocalized exclusively with PLP1 expression in only a subset of myelinated axons (Supplemental Figure 5). As anticipated, ICI of MS#30 and ON#34 into PLP^y/–^ EGFP-PLP mice did not cause the complement-mediated oligodendrocyte (OG) cell death observed in male WT animals (Figure 4B). Because MS#11 does not bind to fixed CNS tissue (data not shown), we could not evaluate binding to PLP^y/+^ and PLP^y/–^ myelin by immunostaining.

### PLP1-transfected cell lines do not fully capture high-affinity epitopes formed on purified myelin.

**Figure 5:**
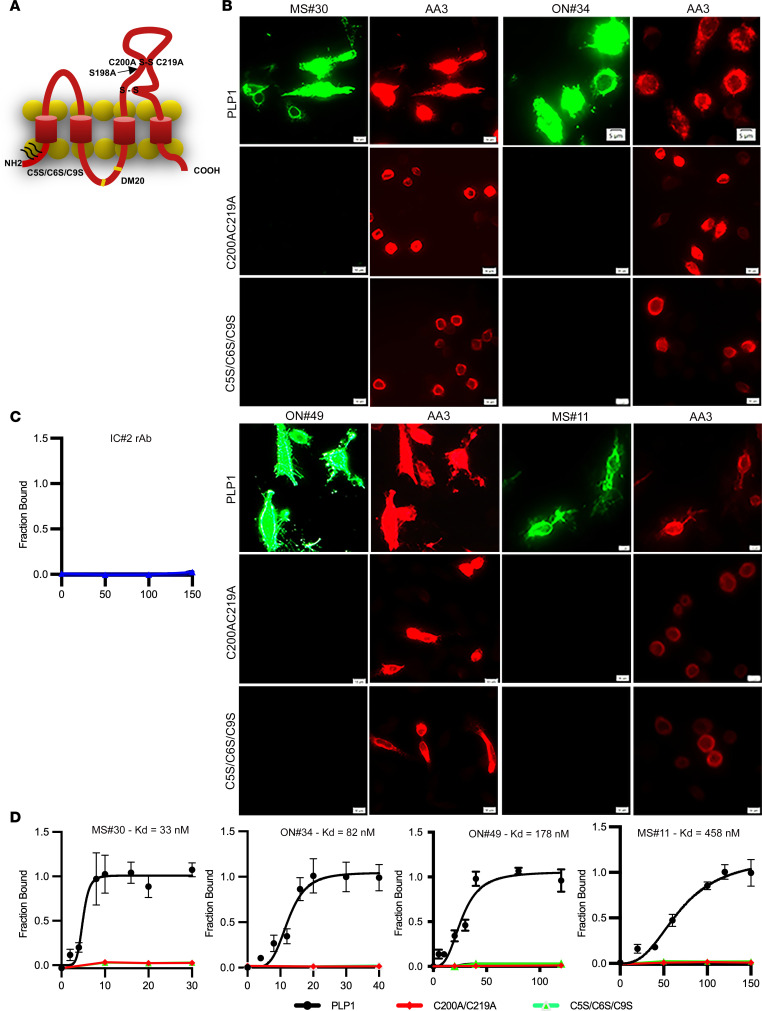
Myelin-specific MS rAbs recognize complex PLP1 antigens. (**A**) Schematic of extracellular and intracellular mutations introduced into PLP1. (**B**) Immunofluorescence images of MS#30 (20 µg/ml), ON#34 (40 µg/ml), ON#49 (50 µg/ml), and MS#11 (100 µg/ml) rAb binding to live cells expressing WT PLP1 or PLP1 containing mutations in the second extracellular domain (C200A/C219A) or N-terminal cysteines (C5S/C6S/C9S). Scale bars: 5 μm. (**C**) Quantitative assay shows absence of IC#2 rAb binding (μg/ml) to live cells expressing WT PLP1. (**D**) Quantitative binding (mean ± SEM) of MS#30, ON#34, ON#49, and MS#11 rAbs (μg/ml) to cells expressing WT or mutated PLP1. Apparent *K*_D_ are reported for each experimental curve.

To better delineate the PLP1 epitope, we quantified binding of individual pathogenic MS rAbs to live CHOK1 cells transfected with PLP1, the DM20 splice variant, or with targeted PLP1 point mutations. These point mutations alter the outer disulfide loop of the large second extracellular domain of PLP1 (C200A/C219A), a putative loop 2 extracellular palmitoylation site (S198A), or N-terminal intracellular cysteines (C5S/C6S/C9S), required for sorting of PLP1 to myelin-like membranes (Figure 5A) (17, 18).

Each pathogenic MS rAb bound to live PLP1-transfected cells in a concentration-dependent manner, but none bound to PLP1-containing mutations C200A/C219A or C5S/C6S/C9S (Figure 5, B and D). No binding to PLP1^+^ cells was observed with the IC rAb IC#2 (Figure 5C). Calculation of apparent equilibrium *K*_D_ showed that MS#30 had the highest (17 ± 16 nM) and MS#11 the lowest (358 ± 88 nM) affinity binding to PLP1-expressing CHOK1 cells. The extracellular C200A/C219A and intracellular C5S/C6S/C9S mutations abolished binding to both live and PFA-fixed cells (data not shown). Because PLP1 expression is highly susceptible to individual point mutations that cause retention in the endoplasmic reticulum, interpreting the effects of individual point mutations on live-cell MS rAb surface binding can be confounding. Mutation of cysteine residues forming the outer disulfide loop, however, was found to be dispensable for cell-surface expression of PLP1 (19). Inhibition caused by mutation of N-terminal intracellular cysteines (C5S/C6S/C9S) indicated that S-palmitoylation may be critical at some stage in epitope formation, although we cannot distinguish whether this effect is on cell-surface transport, epitope assembly, or both. We observed no obvious changes in MS rAb staining of cells expressing the DM20 isoform or the S198A point mutation (data not shown).

**Figure 6:**
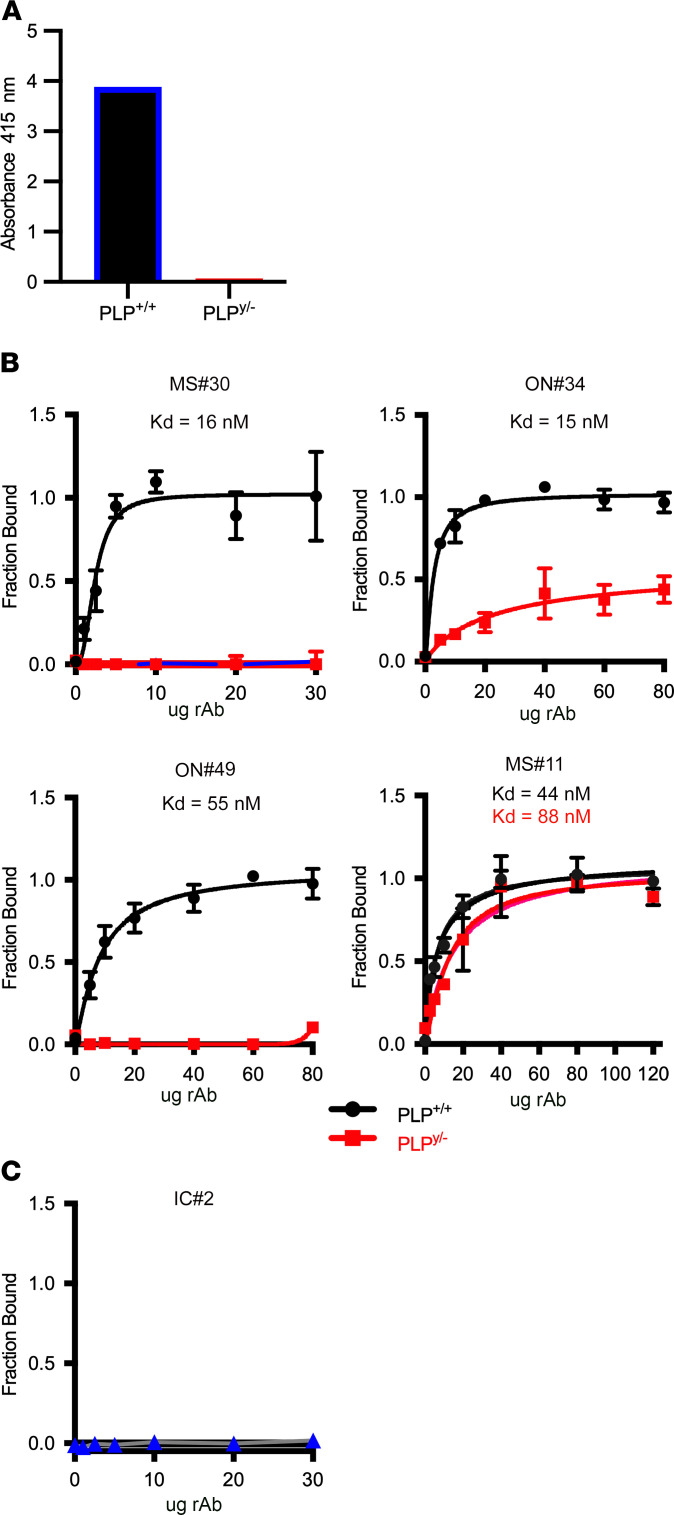
Binding of MS rAbs to purified myelin. (**A**) ELISA demonstrating differential binding of control PLP1-specific AA3 mAbs to PLP1 WT (+/+) and null (y/–) myelin. (**B**) MS rAb binding (mean ± SD) to purified myelin from WT and null PLP1 animals was measured by ELISA. (**C**) IC rAb IC#2 binding curve to WT myelin is shown as a negative control.

We also quantified binding of rAbs to purified myelin from WT and PLP1-null animals by ELISA. Specificity of this assay for the presence or absence of PLP1 protein was confirmed by the differential binding of the control PLP1-specific mAb AA3 (Figure 6A).

MS rAbs, excluding MS#30, showed a 4- to 10-fold increase in binding affinity to WT myelin when compared with PLP1-transfected cells (Figure 6B and Table 1). No binding to purified myelin from PLP1 WT animals was observed with the IC rAb IC#2 (Figure 6C). While ON#34 showed weaker but measurable binding to myelin from PLP^y/–^ null animals, MS#11 bound with similar affinity to both WT and PLP1-null myelin. Taken together, these data suggest that the myelin-binding target of MS rAbs is a PLP1 membrane complex that requires factors other than PLP1 to generate high-affinity epitopes.

### Myelin-enriched glycolipids and cholesterol affect binding of pathogenic MS rAbs to PLP1-transfected cells.

Because PLP1 is targeted to the myelin membrane in raft-like domains enriched in glycolipids and cholesterol, (20) we postulated that increased expression of raft-associated glycolipids galactocerebroside (galc) and sulfatide, which are enriched in myelin, might influence epitope formation in PLP1-transfected cell lines. CHO cells were transfected with plasmid vectors expressing full-length cDNAs for UDP-galactose ceramide galactosyltransferase (CGT) (galc biosynthesis) and CGT plus cerebroside sulfotransferase (CST) (sulfatide biosynthesis). Galc and sulfatide expression were confirmed using mAbs against the oligodendrocyte cell-surface developmental markers O1 and O4 (Supplemental Figure 6A) (21, 22). Neither O1 nor O4 bound to the surface of live, untransfected CHO cells. Transfection of CGT, but not CST, produced multiple O1^+^ cells, whereas positive O4 staining required the coexpression of both CGT and CST (Supplemental Figure 6, A and B). MS rAbs did not bind to O4^+^ CHO cells in the absence of PLP1 (Supplemental Figure 6C).

**Figure 7:**
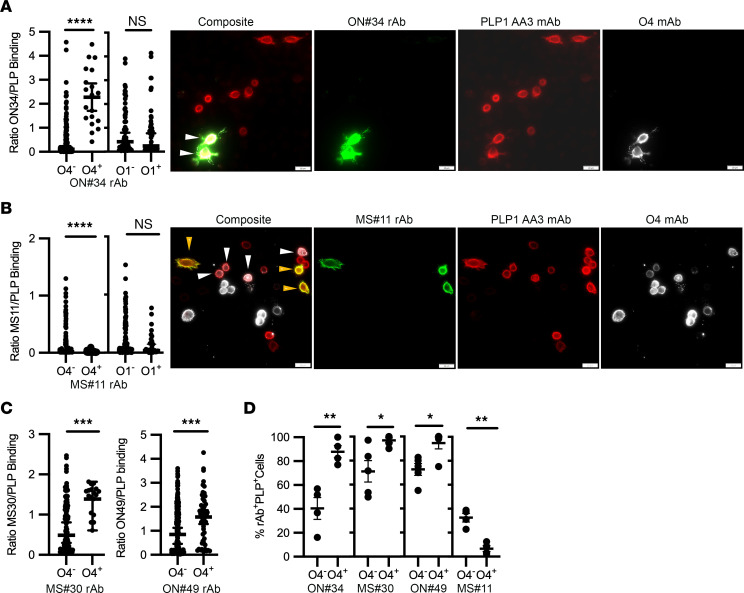
Sulfatide and cholesterol levels modify binding of MS rAbs to PLP1-expressing cells. (**A**) Quantitation and representative images of ON#34 rAb binding to PLP1^+^ CHOK1 cells coexpressing sulfatide (O4) or galc (O1). Scale bars: 20 μm. The binding intensity ratio of ON#34 rAb (green, G) to PLP1 mAb (red, R) (G/R ratio) is plotted (median ± 95% CI) for single PLP1^+^ cells (*n* = 19 PLP1^+^O4^+^ and 131 PLP1^+^O4^–^ cells; *n* = 52 PLP1^+^O1^–^ and 65 PLP1^+^O1^+^ cells) and significance established using Welch’s *t* test. White arrowheads on images identify ON#34^+^O4^+^PLP1^+^ cells. (**B**) Representative images and quantitation of MS#11 rAb binding to PLP1^+^ CHOK1 cells coexpressing sulfatide or galc. The ratio of MS#11 (green) to PLP (red) is plotted (median ± 95% CI) for PLP1^+^ cells binned according to O4 (*n* = 161 PLP1^+^O4^–^ and 45 PLP1^+^O4^+^ cells) or O1 expression (*n* = 145 PLP1^+^O1^–^ and 34 PLP1^+^O1^+^ cells) and significance established using Welch’s *t* test. White arrowheads identify MS#11^–^O4^+^PLP1^+^ and gold arrowheads MS#11^+^O4^–^PLP1^+^ cells. (**C**) The binding ratios of MS#30 (green) or ON#49 (green) to PLP (red) are plotted (median ± 95% CI) for PLP1-expressing CHOK1 cells binned for O4 expression (MS#30: *n* = 127 PLP1^+^O4^–^ and 18 PLP1^+^O4^+^ cells; ON#49: *n* = 249 PLP1^+^O4^–^ and 50 PLP1^+^O4^+^ cells). Significance was established using Welch’s *t* test. (**D**) Quantification (mean ± SEM) of the percentage of PLP1AA3-expressing cells positive for MS rAb staining (determined by G/R ratio) according to the visual presence or absence of coincident O4 mAb staining. Values represent measurements from 4 to 5 independent experiments per MS rAb. *P < 0.05; **P < 0.01; ***P < 0.001; **** *P* < 0.0001, Welch’s *t* test.

We transfected cells with PLP1 following separate transfections with CGT alone or CGT and CST. Sulfatide (O4^+^ staining), but not galc (O1^+^ staining), expression had a strong and significant positive effect on the mean G/R binding ratio of rAb ON#34 (Figure 7A). In contrast, rAb MS#11 showed reduced binding in the presence of sulfatide (Figure 7B), but not by the expression of galc. As with ON#34, sulfatide expression also enhanced the binding of rAbs MS#30 and ON#49 to PLP1^+^ cells (Figure 7C). Sulfatide expression increased the fraction of PLP1^+^ cells bound by rAbs ON#34, ON#49, and MS#30 and reduced the fraction of cells recognized by rAb MS#11 (Figure 7D).

**Figure 8:**
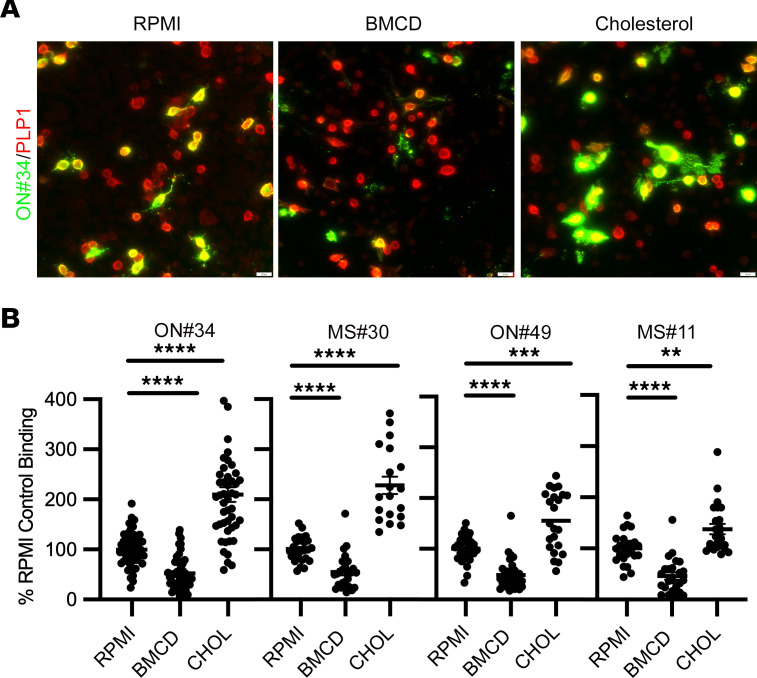
Cholesterol affects binding of MS rAbs to PLP1-expressing cells. (**A**) Representative images of ON#34 rAb binding to PLP1-transfected CHOK1 cells following 30-minute treatment with vehicle (RPMI), 5 mM BMCD, or 5 mM ΒMCD preloaded with cholesterol (CHOL). Scale bars: 20 µm. (**B**) Quantitation (mean ± SEM) of background-corrected ratios of MS rAb to PLPAA3 signal normalized to RPMI control binding. Data are represented as ratios of replicate images obtained from more than 3 independent transfection experiments. Significance was determined using ANOVA with Dunnett’s correction for multiple comparisons. **P < 0.01; *** *P* < 0.001; **** *P* < 0.0001.

We next assessed the influence of cholesterol, also enriched in the myelin sheath, on MS rAb PLP1 epitope formation and stability (Figure 8). Treatment of live PLP1-transfected cells for 30 minutes with 5 mM B-methyl-cyclodextrin (BMCD), a compound that rapidly sequesters cholesterol and disrupts both membrane lipid rafts and tetraspanin-enriched domains (23, 24), reduced the binding of all the MS rAbs by approximately 2-fold when normalized to cells treated with vehicle alone (images are shown for ON#34 rAb). In contrast, adding cholesterol to transfected PLP1 cells via treatment with 5 mM BMCD saturated with soluble cholesterol improved binding to a similar degree. To ensure that loss of binding following BMCD treatment was not due to the selective death of PLP1^+^ cells, we immediately stained cells after treatment with DRAQ7 dye followed by our standard immunostaining protocol. There were no significant differences in PLP1^+^ cell viability between RPMI- (98.1% ± 3.2%), BMCD- (97.7% ± 10.7%), and cholesterol-treated (98.9% ± 5.1%) cells.

To better interpret the independent or synergistic effects of lipids on PLP1 complex epitope formation, we generated a stable cell line in parental HEK293 (HEKP) cells expressing both CGT and CST cDNAs (clone HEKPE7). This cell line showed a clear differential positive staining with both O1 and O4 mAbs (Supplemental Figure 7). ON#34 rAb binding was enhanced more than 5-fold to PLP1-transfected HEKPE7 cells (Supplemental Figure 7), mirroring results reported in Figure 7A. Treatment of HEKPE7 cells with cholesterol further enhanced PLP1 binding to the E7 cell line (Supplemental Figure 7). A more modest but unexpected increase in MS#11 binding was observed in the E7 cell line that was also accentuated by cholesterol treatment (Supplemental Figure 7). The contradictory effects of sulfatide expression on MS#11 rAb binding in CHOK1 (Figure 7) and HEKPE7 cells may be due to steric hindrance from the O4 and secondary anti-IgM Abs used to identify sulfatide expression in transiently transfected CHOK1 cells.

### Detection of Abs to PLP1 complexes in MS and inflammatory control CSF.

**Figure 9:**
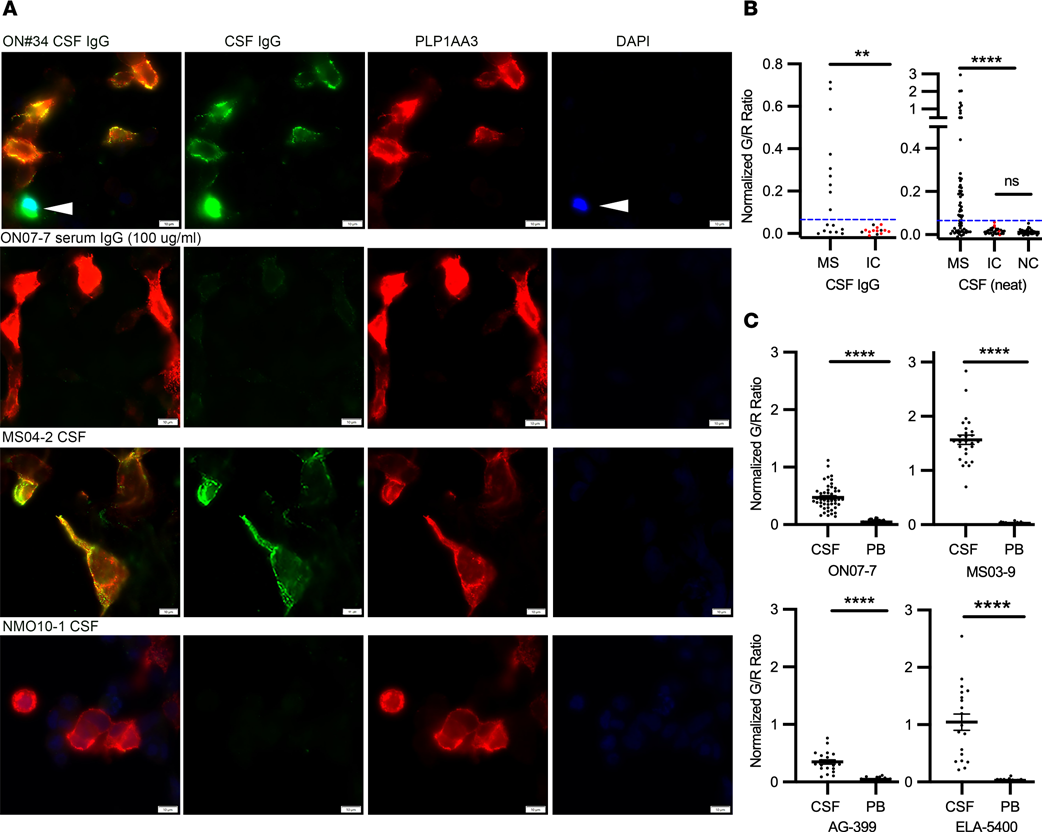
Detection of conformational PLP1-specific Abs in MS and control CSF. (**A**) PLP1 and human IgG immunofluorescence on live PLP1-transfected HEKPE7 and cholesterol-treated cells 24 hours after transfection using CSF IgG (100 µg/ml), CSF neat, or serum IgG from MS or inflammatory control patients. Following cholesterol treatment, live-cell cultures were treated with DAPI (arrowheads) to identify dead cells. Scale bars: 20 µm. (**B**) Normalized and background-corrected mean ratios of green/red immunofluorescence signal (G/R) to single or small clusters of PLP1-transfected cells are shown for cohorts of clinically definite MS (*n* = 79), infectious (red circles) and noninfectious inflammatory neurologic controls (IC, *n* = 45), and noninflammatory neurologic controls (NC, *n* = 39) using either purified CSF IgG or CSF neat. Blue dashed lines indicate values 3 SDs above the mean of IC binding and distinguished positive from negative PLP1 binding. No significant differences in the mean G/R ratio were observed in the populations comprising IC CSF IgG (G/R = 0.015 ± 0.015), IC CSF (G/R = 0.016 ± 0.013) and noninflammatory neurologic controls (G/R = 0.013 ± 011). Fisher’s exact test was used to compare distributions between MS and control patients for each cohort; **P < 0.01; ****P < 0.0001. (**C**) Normalized ratios of green/red immunofluorescence signal (G/R) for PLP1-transfected cells incubated with the same IgG concentrations of serum or CSF from 4 PLP1^+^ MS patients. The ratio of mean CSF and serum-binding titers were greater than 8.2 for each patient, indicative of intrathecal synthesis of PLP1 complex–specific Abs. Statistical comparisons of replicate measurements from 2 to 3 independent transfections were made using Welch’s *t* test. ****P < 0.0001. Data are represented as means and SD.

Developing a specific screening assay to detect conformational PLP1-specific Abs in MS patient CSF and serum was challenging due to the reduced sensitivity of live-cell assays and the reduced specificity of purified myelin ELISAs. Neither undiluted ON07-7 nor MS04-2 CSF (Supplemental Table 1), the source of pathogenic PLP1-specific rAbs, showed visual staining of live PLP1-transfected cells (data not shown) in the absence of glycolipids/cholesterol. However, staining of live PLP1-transfected HEKPE7 cells treated with cholesterol yielded enhanced assay sensitivity; both MS patient ON07-7 CSF IgG (100 µg/ml) and MS04-2 CSF displayed a robust and unambiguous positive surface staining of PLP1-transfected cells that was not observed with NMO CSF (Figure 9A). Dead cells were identified and omitted from consideration by live-cell DAPI staining following cholesterol treatment. DAPI^+^ cells typically, but not universally, showed a bright and smeared cytoplasmic staining with patient CSF that was readily identified and morphologically distinct from the punctate surface staining observed for live PLP1^+^ cells (Figure 9A). Using purified CSF IgG, we visually identified positive PLP1-specific cell staining in 9 of 17 MS and 0 of 14 (CNS infectious and noninfectious) inflammatory (*n* = 13) or noninflammatory (*n* = 1) control CSF IgG samples. We further quantified and normalized the average CSF IgG/PLP1 (G/R) binding ratios (Supplemental Figure 8A) on individual or small clusters of PLP1^+^ cells per CSF sample as visualized in Supplemental Figure 8A and Figure 9B. Using a cutoff of G/R binding ratio greater than 3 SDs above the mean average of control CSF IgG samples, we confirmed positive staining on the same set of 9 positive MS CSF samples visualized by specific PLP1^+^ cell staining.

We next assayed a separate and distinct cohort of MS and control CSF from both our laboratory sample bank and from 78 masked samples provided by the Rocky Mountain MS Center Biorepository. This data set included an additional 63 clinically verified MS CSF samples, 29 inflammatory, and 38 noninflammatory brain tumor, headache, or migraine controls (Supplemental Table 2). PLP1^+^ cell-surface staining in the live-cell assay was highly specific for MS CSF samples, with 58.7% of MS and 0% of inflammatory and noninflammatory control patients showing positive staining both visibly and by G/R binding ratios (Figure 9C). Representative images of CSF samples with weak (MS04-4, G/R ratio = 0.089), intermediate (MS09-1, G/R ratio = 0.56), robust (MS93.1, GR ratio = 0.96), and negative HEKPE7 binding are presented in Supplemental Figure 8B. Altogether, anti–PLP1 complex Abs were identified in the CSF of 46 of 80 different MS patients (57.5%). The frequency of MS patients with positive anti–PLP1 complex Abs was significantly increased relative to controls whether using neat CSF or purified CSF IgG (Figure 9B).

We compared anti–PLP1 complex–specific antibody titers from paired CSF and peripheral blood of 4 positive MS patients (Figure 9C). PLP1-specific binding was significantly lower and barely observable in the peripheral blood of each patient. The ratio of the mean CSF IgG– and serum IgG–binding titers was 8.2 or greater for each MS patient, indicating intrathecal production of PLP1-specific Abs (Figure 9C). Longitudinal sampling of patients ONO7-7 (Supplemental Figure 8, MS09-1) and MS05-3 (Supplemental Figure 8, MS07-8) identified the continued presence of PLP1-specific IgG in CSF 18 months or more after initial diagnosis.

Anti–PLP1 complex Abs were identified in both relapsing and progressive MS patients. Nine out of twelve CSF samples from patients with progressive forms of MS were positive for Abs against membrane PLP1 complexes (5/5 secondary progressive and 4/7 primary progressive patients). We produced and tested 8 rAbs from primary progressive MS patient MS02-19, who demonstrated a high CSF PLP1–specific antibody titer (G/R ratio = 1.3, Supplemental Figure 8). None of the 8 MS02-19 CSF-derived rAbs bound to PLP1-transfected CHOk1 or HEKPE7 cells after cholesterol treatment (Supplemental Table 3). Similarly, CSF from RRMS patients MS03-1 and MS05-3 was positive for PLP1 staining, even though a comprehensive testing of rAbs derived from clonal plasmablast populations obtained at the time of lumbar puncture failed to stain PLP1-transfected cells (Supplemental Table 3 and Supplemental Figure 8).

## Discussion

We have identified myelin PLP1 as a core component of an antigen complex recognized by myelin-specific MS CSF plasmablast clones. Myelin-specific MS rAbs derived from clonally expanded CSF plasmablasts bind to the surface of live PLP1-transfected cells and stain white matter tracts in WT but not PLP1-null animals. One member of this group, rAb MS#11, did not bind myelin in CNS tissue by immunostaining, but did bind to live PLP1-transfected cells and to myelin from PLP1 WT and null animals by ELISA. In the presence of HC, PLP1^+^ rAbs induce rapid complement-dependent oligodendrocyte cell death in organotypic slice cultures (15). Following ICI with HC into mouse brain, PLP1 complex–targeting rAbs cause a striking loss of EGFP^+^ cell bodies and processes, recruitment of CD68^+^ phagocytes, terminal complement complex deposition on myelin sheaths, and phagocytic engulfment of oligodendrocyte cell bodies (Figure 1 and Supplemental Figures 1 and 3). Lesion formation is absent following injection of rAbs MS#30 and ON#34 with HC into PLP1^y/–^ animals, confirming PLP1 as a bona fide pathogenic antigenic target that provides a putative identity to the IgG demyelinating activity described in some MS patients (25–27).

An unexpected observation in the in vivo model is the transient loss of astrocytes within lesions that was not observed in ex vivo spinal cord and cerebellar slice culture models (14, 15). Astrocyte recovery is rapid, with activated astrocytes returning to areas of CD68^+^ cell infiltration until at least 14 days after injection. The mechanism driving early astrocyte loss is unknown, but could be promoted by bystander complement injury from myelin to adjacent astrocytes, as recently observed from astrocytes to oligodendrocytes and neurons in lesional models of NMO (28–30). Whether astrocyte loss in our model is duplicative of the loss of astrocytes reported in hyperacute early demyelinating MS lesions remains to be determined (31).

The nature of antigen recognition is complex. Only a fraction of transfected PLP1^+^ cells are stained by PLP1-specific MS rAbs, and staining is sensitive to organic fixation and plasma membrane composition (Figures 5–8 and Supplemental Figure 4). Access to intracellular PLP1 in PFA-fixed and permeabilized cells does not improve binding, indicating that PLP1 protein constitutes a key component of a conformationally sensitive surface-membrane antigen assembled posttranslationally. These structural requirements explain the failure to detect PLP1 immunoreactivity in our initial survey of MS rAb specificities, as detection was obscured by methods used for fixation and tissue processing (13).

PLP1 is a distant member of the tetraspanin family of proteins, molecules known for molecular organization at the cell membrane (32). We hypothesize that posttranslational tertiary changes, such as assembly of PLP1 into multimolecular and lipid-rich surface domains, are an essential step in epitope formation that is limiting in nonlineage and immortalized cell lines. Supporting this view, we found that binding affinities of most PLP1-positive rAbs are reduced approximately 4- to 10-fold in transfected cells when compared with purified myelin, mutation of intracellular N-terminal palmitoylation sites (C5S/C6S/C9S) abrogates epitope formation, and expression of sulfatide and the modulation of cholesterol levels significantly augments binding to PLP1^+^ cells. The idea of PLP1-anchored membrane domains is further illustrated by the binding of MS#30 to discrete regions on the surface of oligodendrocyte processes and myelin in live cerebellar slice cultures (15). In differentiating oligodendrocytes, PLP1 associates with cholesterol and is delivered to the myelin sheath in lipid rafts enriched in both cholesterol and glycolipids (17, 20). Recreating this microenvironment in transfected CHO and HEKPE7 cell lines appears to drive PLP1 epitope formation. One caveat that hinders full interpretation of live-cell staining data is the effect of some mutations on PLP1 transport to the cell surface complicating distinctions between PLP1 surface expression and epitope assembly. In a previous study, disruption of the outer disulfide loop in the large PLP1 extracellular domain via mutation of C200 and C219 knocked out binding to the conformational PLP1-specific mAb O10, but not PLP1 transport to the cell surface (19). The C200A/C219A mutation in our studies also abolished all binding to PLP1, and we speculate that pathogenic myelin-specific MS rAbs may replicate the intricate and native antigen specificity originally defined by the O10 mAb, which was generated via immunization of animals with corpus callosum homogenate (33). The essential role of N-terminal cysteines in epitope formation is more difficult to parse and could be related to membrane transport, as they are a sorting determinant to myelin (18, 34), but could also facilitate protein interactions within the cell membrane that play a role in the insertion of tetraspanins into membrane domains. Both sets of mutations define domains essential for epitope formation, as binding is eliminated in both live and PFA-fixed cells. Another key and intriguing question raised by the unusual properties of MS#11 rAb is the contribution of additional unknown molecules to PLP1 complex formation. While rAb ON#34 shows lower binding affinity to myelin from PLP1-null animals, rAb MS#11 binds to PLP1 WT and null myelin with almost equal affinity (Figure 6), is sensitive to all fixation methods, and does not stain PFA-fixed brain tissue. Akin to the other PLP1-specific rAbs, rAb MS#11 binding is modulated by cholesterol; however, distinct from other myelin-specific MS rAbs, rAb MS#11 binding to PLP1-transfected cells is either blocked or only modestly enhanced by sulfatide expression, depending on experimental conditions (Figure 7 and Supplemental Figure 7). To explain this disparate behavior of PLP1-reactive rAbs, we propose a working model that infers integration of PLP1 into lipid-rich complexes with an unknown binding partner and suggests that differences in rAb specificity are a result of the relative contribution of individual molecules to epitope formation and recognition. In this scenario, we envision rAb MS#30 binding primarily to PLP1 and rAb MS#11 binding to the hypothetical binding partner.

Although CSF IgG OCBs remain a useful diagnostic biomarker of disease in MS, attempts to identify a uniform set of antigenic targets and determine their relationship to disease pathology has led to a confounding and contradictory body of literature. Results vary according to the screening protocol, immunologic assay, and source of antibody. The plethora of identified antigenic targets obtained screening MS CSF IgG include viruses, bacteria, myelin proteins, intracellular proteins, glycolipids (35–37), and the neuronal adhesion molecule contactin-2 (38). Some specificities are likely attributable to both the fidelity of the immune assay and the polyclonal background response observed in MS and other CNS infectious diseases. In studies of B cell clones recovered from SSPE brain tissue or CSF, 60%–75% of rAbs bound to measles virus antigens, (39, 40), whereas in a population study of mumps meningitis patients, 90% of subjects had CSF OCBs directed against mumps virus, but 40% also had additional bands directed against other viral antigens (measles, herpes simplex virus [HSV], or rubella virus) (41). Polyclonal activation of long-lived memory cells has been observed following primary immunization (42) and may arise through bystander T cell activation or engagement of Toll-like receptors in a proinflammatory B cell environment (43). In active NMO patients with AQP4 autoantibodies (AQP4-IgG), there is a prominent expansion of CSF plasmablast clones in the absence of corresponding CSF OCBs (44, 45) and only 60% of infiltrating plasmablast clones are targeted against conformational AQP4 epitopes (44).

Recently, it has been postulated that OCBs are directed against intracellular debris (35, 46). Although we agree that B cell responses to cellular debris may occur following CNS damage (47, 48), a global consensus regarding the specificity of select IgG bands and their relationship to ongoing disease processes should be tempered by consideration of the source of Abs and the screening strategies employed for antigen detection. Brandle et al. (46) combined proteomic and transcriptomic data to construct full-length rAbs from abundant CSF oligoclonal IgG. In contrast, our study generated rAbs from clonally expanded CSF plasmablast clones (13). While there is overlap between the CSF plasmablast/plasma cell transcriptome and CSF immunoglobulin proteome/OCBs, the relationship is likely complex and nonlinear based on the life span, location, and maturation of CSF B cell clones, disease activity, MS treatment, single-cell sampling, and methodologic sensitivities. While CSF IgG OCBs are markedly stable (49, 50), the appearance and prominence of individual CSF B cell clones fluctuate considerably (51). As demonstrated herein, for some MS subjects producing anti–PLP1 complex Abs in the CSF, identification of their cognate PLP1-specific B cell clones within the CSF pool appears stochastic and bears no obvious relationship to corresponding CSF antibody titers. Because PLP1-reactive Abs target complex epitopes that are dependent on preserved tertiary and quaternary structure, target screening with high-throughput protein arrays or phage-display expression libraries will likely miss this intricate specificity. Indeed, rAb MS#30 does not bind to any of the myelin proteins (our unpublished observations), peptides, or lipids deposited onto myelin microarrays (52). This again draws parallels to the structural requirements promoting binding of AQP4-IgG to AQP4 tetramers and orthogonal arrays in NMO (53, 54), in which the assembly of multimeric rAb clusters on complex AQP4 orthogonal arrays drives C1q binding and activation of the classical complement pathway (55). An MS CSF–derived rAb to EBV nuclear antigen-1 was recently shown to be crossreactive with an intracellular peptide of the CNS protein GlialCAM, suggesting an immunologic link between MS and EBV infection (36). None of the pathogenic PLP1-reactive MS rAbs described herein bound to cells expressing GlialCAM protein (our unpublished observations).

The detection of corresponding PLP1-specific Abs in MS patient CSF is greatly facilitated by sulfatide and cholesterol (Figure 9), which primarily enhance epitope formation. Identifying disease-relevant Abs in serum or plasma is even more difficult due to lower titers; the ratio of the mean CSF and serum IgG titers from 4 different patients, including ON07-7, indicates a strong intrathecal production of PLP1-reactive Abs. This does not suggest that a serum-based assay cannot be developed, but that it will likely require lower dilutions than the approximate 1:100 dilution of sera/plasma used to compare CSF and peripheral blood binding. Recognizing additional molecular components of PLP1 complexes that can be manipulated to improve affinity of the live-cell assay may also improve the ability to observe PLP1 antibody responses in blood. We observed PLP1-reactive Abs in 58% of MS CSF samples, including the very onset of disease. Two PLP1-reactive rAbs, ON#34 and ON#49, were generated from CSF plasmablasts recovered from patient ON07-7 at the time of her first clinical attack; all brain MRI lesions evident at the time of presentation were new and enhancing. Given the high specificity of our assay, as evidenced by the absence of PLP1 complex Abs in more than 80 combined inflammatory and non-MS control CSF samples, the detection of conformational immunoreactivity to PLP1 membrane complexes in CSF or serum may provide a new diagnostic tool for MS.

Recently, a serum response to the large extracellular loop of PLP1 has been reported in an HLA-restricted subgroup of MS patients using a conformational peptide ELISA (56). The putative relationship between these serum PLP1 peptide–binding IgGs and the PLP1 complex–specific MS rAbs described here remains unclear, but there is likely some overlap, as both IgG species are sensitive to disruption of the outer disulfide bonds within the large extracellular loop. Together, the presence of these autoantibodies in MS patients reinforces the notion of a pathogenic disease-relevant immune response that is at least partially directed against PLP1. PLP1 may also prove important for the recruitment of other target molecules to the cell surface that contribute to the formation of high-affinity epitopes. Given the importance of membrane lipids and cholesterol for PLP1 complex autoantibody binding, the presence of anti-glycolipid Abs in MS patients may play a role in modulating the antibody response to PLP1 complexes (37).

In conclusion, we postulate that myelin PLP1 complexes are one target of a larger B cell immune response in MS. Autoantibodies against PLP1 likely contribute to CNS injury in a subset of MS patients, perhaps those presenting with type II white matter lesion pathology (1) and type III cortical demyelination, and operate at the earliest stages of disease. Conformational PLP1-specific Abs now offer a potential biomarker for disease diagnosis that could facilitate MS diagnosis and improve prognosis and treatment. Pathogenic monoclonal Abs will also facilitate disease-relevant models to untangle the neuroscience of lesion formation and repair (15, 57).

## Methods

### Animals.

Animals used in this study included C57BL/6 transgenic mice expressing EGFP (PLP-EGFP) in oligodendrocytes driven by the PLP1 promoter, (16) PLP1-null mice crossed into a C57BL/6 PLP-EGFP background (58), and Swiss Webster Tg(Aldh1l1-EGFP,-DTA)D8Rth (59).

### Monoclonal human rAbs.

CSF and plasma were obtained from MS (*n* = 79), inflammatory control (*n* = 45), and control patients (*n* = 39). MS CSF–derived monoclonal rAbs used in this study were constructed from expanded CSF plasmablast clones identified in the CSF of relapsing-progressive (MS04-2), relapsing-remitting (MS05-3), primary progressive (MS02-19), and clinically isolated patients (Supplemental Table 1) who subsequently progressed to clinically definite MS (ON07-7 and MS03-1). IC rAbs were generated from a chronic meningitis patient CSF plasmablast clone (IC#2) and SSPE CNS tissue (2B4 rAb). Amplification of VH cDNA from sorted CSF plasmablasts included sufficient C-region sequence information to identify the IgG subclass (60). All rAbs were expressed as full-length bivalent human IgG1 Abs containing a C-terminal Flag epitope that matched their subclass in vivo expression except for MS#30, which utilized the IgG2 subclass in CSF plasmablasts. rAbs were produced using a dual vector transient transfection system in EXPI 293 cells (Thermo Fisher Scientific) and purified with protein A–sepharose (Sigma-Aldrich) as previously described (13, 44). Banked MS and control patient CSF and sera IgG were also purified with protein A–sepharose. Inflammatory control CSF or purified CSF IgG was obtained from multiple CNS inflammatory infectious and autoimmune diseases. CNS inflammatory infectious diseases included 2 cases of SSPE, 2 cases of neurosyphilis, 8 cases of viral or cryptococcal meningitis, 1 case of Whipple’s disease, and 2 cases of varicella zoster virus–associated (VZV-associated) encephalitis or vasculopathy. Verified CNS autoimmune disorders included 1 case of Sjögren’s syndrome with neurologic complications, 4 cases of NMO, 2 cases of myelin oligodendrocyte glycoprotein-IgG–associated disease, and 3 cases of CNS paraneoplastic disease. CNS inflammation of unknown cause included 4 cases of neurosarcoidosis, 1 case of chronic meningitis, 1 case of vasculitis, 1 case of encephalitis, 1 case of acute disseminated encephalomyelitis, and 8 cases of isolated CNS white matter disease. Noninflammatory CNS controls included 1 patient with a CNS brain tumor, 1 patient with a CNS B cell malignancy, and 37 patients with headache or migraines.

### Primary and secondary Abs.

Primary Abs used in this study included biotinylated rat anti-mouse CD68 mAbs (clone FA-11, Bio-Rad), mouse anti-GFAP mAbs (clone G-A-5, MilliporeSigma), rabbit anti-GFAP (MilliporeSigma), mouse anti-MOG IgG mAbs (clone 8-18C5, gift of Claude Genain (University of California, San Francisco, California), rabbit polyclonal anti-human C5b-9 IgG (Calbiochem), mouse anti-plp1 IgG mAbs (clone plpc1, Thermo Fisher Scientific), rat anti-PLP1 AA3 IgG mAb hybridoma supernatant (61) targeting an intracellular C-terminus PLP1 epitope (produced in house), mouse O1 and O4 oligodendrocyte antigen hybridoma mAb (21) supernatants (produced in house) (IgM specific), and mouse anti-Flag IgG mAbs (clone M2, Stratagene). Isotype-matched fluorescent secondaries included Alex Fluor 488–conjugated donkey anti-human Fc fragment–specific IgG (Jackson ImmunoResearch), Alex Fluor 488–conjugated goat anti-human IgG (Invitrogen), Alexa Fluor 594–conjugated donkey anti-rat IgG (Jackson ImmunoResearch), Alexa Fluor 647–conjugated streptavidin (Invitrogen), Alexa Fluor 647–conjugated goat anti-mouse IgM (Invitrogen), Alexa Fluor 594–conjugated goat or donkey anti-mouse IgG (Invitrogen), HRP-conjugated goat anti-human IgG (Pierce Chemical Co.), and HRP-conjugated goat anti-rat IgG (Pierce Chemical Co). Secondary Abs were used at 1:800–1:1,000 dilutions.

### ICI of MS rAbs and HC.

MS and IC rAbs were injected into right hemisphere thalamic regions (Bregma: *x* = 1 mm, *y* = –1.6 mm, *z* = –3.7 mm) of PLP-EGFP mice (16). Male and female adult animals were used as available for these studies. Injections (5 µl) contained 5 µg rAb ± 20% HC (Complement Technology), Monastral blue dye, and PBS, pH 7.4. Solutions were injected through a Hamilton 33-gauge needle at an automated rate of 0.5 μl per minute. Animals were perfused with 4% PFA at 72 hours after injection; brains were then removed, post-fixed overnight in 4% PFA, and cryoprotected overnight in 20% sucrose at 4°C. Brains were embedded in OCT freezing media, and the approximate injection area identified by Monastral blue dye. Serial coronal sections (10 µm, 40–50 sections) were cut across the block, numbered, and placed in consecutive pairs onto adhesive glass coverslips until tissue sections contained no dye. Sections were imaged without mounting media or coverslips for oligodendrocyte cell loss (EGFP^+^ cell bodies) and then were stored at −80°C for further histochemical studies.

### Expression vectors.

Full-length human PLP1 and MOG cDNAs were originally cloned into the pCEP4 expression vector as described (13). For most expression experiments, PLP1 was subcloned into the pCDNA 3.1 expression vector. Double-stranded DNA G blocks encoding the DM20 isoform and targeted PLP1 mutations C200A/C219A, S198A, and C5S/C6S/C9S were synthesized (IDT Technologies) and cloned into pCDNA3.1 for expression in tissue-culture cells. Commercial plasmid vectors were used for the expression of human UDP-galactose CGT (Sino Biological, NM_001128174.1) and murine CST transcript variant 2 (Origene, NM_001177703). Full-length UDP-galactose CGT and CST cDNAs were amplified from the above expression vectors and cloned into the dual pVITRO-1 MCS expression vector (InvivoGen). Plasmid DNAs were grown in *E*. *coli* DH5α and purified using Endo-Free DNA Maxi Kits (QIAGEN).

### Tissue culture and transfections.

CHOK1 cells, HEK293 (HEKE) cells expressing the EBNA-1 gene (Invitrogen), or HEKP cells were used for protein-expression studies. Proteins expressed in cells included PLP1, MOG, and the enzymes UDP-galactose CGT for galc biosynthesis and CGT plus CST for galactosulfocerebroside (sulfatide) biosynthesis. A stable cell line was generated in HEKP cells (HEKPE7) expressing the CST and CGT enzymes by G418 (500 µg/ml) resistance. Single clones were obtained by limiting dilution and assayed for sulfatide production and binding to PLP1-transfected cells to isolate the HEKPE7 cell line.

CHOK1 cells were grown in RPMI medium containing 10% fetal calf serum and supplemented with penicillin/streptomycin. HEKE, HEKP, and HEKPE7 cells were grown in DMEM high glucose containing 10% FCS and penicillin/streptomycin excluding HEKPE7 cells. HEKE and HEKPE7 cells were supplemented with 50 µg/ml and 100 µg/ml G418, respectively. The day before transfection, growing cells were harvested and plated onto sterile 12 mm glass coverslips (Fisher Scientific) previously coated with poly-l-ornithine for CHOK1 and HEKE cells; HEKP and HEKPE7 cells were plated onto coverslips coated with rat collagen 1 (Neuvitro) or Gibco Geltrex solution (Thermo Fisher). Cells were plated at a density of 2.25 to 2.75 × 10^5^ cells per ml and allowed to grow overnight. Cells were then transfected with plasmid DNA (0.4–0.75 µg per coverslip) using the Lipofectamine 3000 Transfection Kit (Invitrogen) according to the manufacturer’s protocol. In experiments where cells were transfected with PLP1, CGT, and CST, separate transfection reactions containing CGT and/or CST plasmid DNA and PLP1 plasmid DNA were prepared. Cells were first transfected with CGT and/or CST prior to transfection with PLP1. No obvious difference in outcome was noted when the time interval between transfection with CGT/CST and PLP1 was varied from 10 minutes to 16 hours. Cells were immunostained at 24 hours after transfection with PLP1.

Manipulation of cholesterol levels occurred at 24 hours after transfection with PLP1. For cholesterol depletion or addition, solutions of 5 mM BMCD or cholesterol-saturated ΒMCD (MilliporeSigma) were prepared in RPMI medium minus FCS. Cell coverslips were incubated with RPMI, ΒMCD, or ΒMCD plus cholesterol at 37°C for 30 minutes and immediately stained with MS rAbs or purified CSF IgG. In some experiments, RPMI-, ΒMCD- , or ΒMCD plus cholesterol–treated cells were immediately incubated with 3 μM DRAQ7 dye in PBS for 5 minutes at 37°C to assess the effects of treatment on cell viability followed immediately by immunostaining.

### Immunostaining.

The protocols used for the immunofluorescent staining of PFA-fixed mouse and human tissue followed published protocols (14). Live-cell staining of transfected cells growing on glass coverslips was performed in filtered PBS-blocking buffer, pH 7.4, containing heat-inactivated 3% donkey serum and 2% BSA. Coverslips were removed and incubated for 5 minutes on ice with cold blocking buffer, followed by a 1-hour incubation at 4°C with rAb diluted in blocking buffer. The rAb concentrations (µg/ml) varied experimentally and by antibody. In initial binding assays to CNS tissue and cDNA-transfected cell lines, rAbs were assayed at 20 to 40 μg/ml. In some experiments, glycolipid-specific mAbs O1 or O4 were included (1:20–1:40 dilution of hybridoma supernatant) with the primary incubation. To assay the binding of MS rAbs to CHOK1 cells transfected with CGT and CST in the absence of PLP1 (Supplemental Figure 6), stainings with MS rAbs and anti-human IgG secondary Abs were performed on live cells followed by PFA fixation and O4 mAb staining. In the set of experiments assessing the effects of glycolipids and cholesterol on rAb binding (Figure 7 and Figure 8), rAbs were used at concentrations nearing maximal binding as determined from quantitative binding assays (Figure 5) for MS#30 (20 µg/ml), ON#34 (40 µg/ml), ON#49 (50 µg/ml), and MS#11 (100 µg/ml). Cells were then washed twice with blocking buffer for 3 minutes per cycle and incubated with isotype-specific fluorescent secondary Abs for 1 hour at 4°C. Cells were washed with cold PBS for 3 cycles and fixed for 15 minutes at room temperature with 4% PFA. After several PBS rinses, coverslips were incubated for 5 minutes in blocking buffer containing 0.3% Triton X-100 followed by a 1-hour incubation at room temperature with PLP1-specific mAbs directed against intracellular PLP1 epitopes using either mouse plp1c (5 µg/ml) or, more commonly, rat anti-PLP1 mAb AA3 (1:100-1:500). Cells were then washed 3 times with PBS and incubated for 1 hour with Alexa Fluor 594 secondary Abs. Cells were again washed with PBS and mounted for microscopy using either ProLong Gold (Invitrogen) or Vectashield Vibrance (Vector Laboratories) mounting medium. Positive binding to PLP1-transfected cells was based on visible fluorescent rAb staining that was restricted to PLP1^+^ cells.

To assess the live-cell binding of MS and control patient CSF to PLP1 complexes, transfections were performed in the sulfatide-expressing HEKPE7 cell line following a 30-minute incubation with 5 mM cholesterol-saturated ΒMCD. Prior to immunostaining, cells were incubated with DAPI (1 µg/ml in PBS) for 3 minutes at room temperature followed by washing with cold PBS blocking buffer. Purified CSF IgG was used at 100 µg/ml and patient CSF was used neat following the addition of 10× PBS blocking buffer. In those few patients with very high CSF IgG concentrations, typically having CNS infections, CSF was diluted to 100 µg/ml. Comparisons of CSF and peripheral blood binding to PLP1-transfected HEKPE7 cells were performed at matching IgG concentrations formulated on clinically reported CSF and peripheral blood IgG values.

### Myelin ELISA.

Myelin was purified from C57BL/6 or Swiss Webster mouse brain using isotonic homogenization and sucrose gradient sedimentation following published protocols (62). Myelin protein was quantified and aliquots stored at –80°C. Myelin was diluted to 20 µg/ml protein in PBS, and 1 µg total protein/well was bound onto high-binding 96-well flat-bottom polystyrene EIA/RIA plates (Costar, Corning Inc.) for 1 hour at 37°C. The myelin suspension was removed, and wells were blocked with 75 µl of filtered 3% BSA in PBS for 1 hour at 37°C. Blocking buffer was aspirated and 50 µl of diluted primary rAbs in 3% BSA blocking buffer were added to individual wells. Plates were incubated for 1 hour at 37°C. Wells were then washed sequentially with TBS pH 7.5 for 1 cycle and TBS containing 0.1% Tween 20 (TBST) for 3 cycles, followed by a final wash with TBS. Each washing cycle was 4 minutes. Secondary antibody solutions (50 µl per well) containing goat anti-human IgG conjugated to HRP (Pierce, 1:800) or goat anti-rat HRP (1:800) were added to each well and incubated for 1 hour at 37°C. Wells were washed as described and binding quantified following the addition of 100 µl of 0.1M citrate buffer pH 4.2 containing 1 mg/ml 2,2′ azino-bis (3-ethylbenzothiazoline-6-sulfonic) diammonium salt (MilliporeSigma) and 0.03% hydrogen peroxide. Plates were incubated for 20 minutes in reduced light at room temperature and optical densities measured at 415 nm using a 96-well plate reader (Bio-Rad). Experimentally, each assay condition was measured in triplicate and each assay condition also included parallel binding of primary Abs to wells not coated with myelin to correct for nonspecific binding.

### Data analysis and statistics.

Following ICI of rAbs and HC, the loss of EGFP^+^ oligodendrocytes was nearly complete and easily visualized within developing lesions. Area of oligodendrocyte cell loss was used to quantify differences in damage caused by IC or MS rAbs. Regions of oligodendrocyte cell loss were measured on paired adjacent sections from every fifth slide until the lesion boundaries were identified. Paired sections showing the largest area of EGFP loss were considered the lesion center and typically aligned to the site of maximum dye deposition. The average of 5 or 6 measurements within 100 µm of the lesion center was quantified as the area of OG cell loss per animal. CD68 cell infiltration was reported as the density of DAPI^+^CD68^+^ cells within the subcortical hemisphere and was calculated using Olympus cellSens Dimension Desktop v2.3 software. Reported cell densities per animal represented the average of 2 independent measurements per animal near the lesion center. Kruskal-Wallis with Dunnett’s correction for multiple comparisons was used to statistically compare lesion loads, and 1-way ANOVA corrected for multiple comparisons was used to assess CD68 cell infiltration between IC and MS rAb injections. Statistical significance (*P* < 0.05) of pairwise comparisons was determined using the Mann-Whitney *U* test.

The binding affinities of myelin-specific rAbs were determined from both live staining of PLP1-transfected CHOK1 cells and from binding to purified myelin by ELISA. Quantitative analysis of antibody binding to live cells was performed on either a Nikon Eclipse E800 (Nikon) or Olympus scanning fluorescence microscope. Captured images (>4 per condition) were uniformly adjusted and green (MS rAb) and red (PLP1 control mAb) channel mean intensities measured using ImageJ software (NIH). The measured green and red channel intensities were corrected for background binding by subtracting the mean binding intensity of each secondary antibody alone. We also generated a binding curve in each experiment to correct for nonspecific binding of IgG1 using IC05-2 #2 (IC#2) rAb. In practice, the binding of nonspecific IgG to PLP1-transfected cells was negligible and could be ignored. The subtracted G/R ratios for each experimental condition were then normalized to the averaged values obtained at saturation binding (Bmax) to yield the fraction bound. Saturation binding curves were plotted using PRISM 10 software and apparent *K*_D_ calculated using specific binding with Hill slope. ELISA optical densities were corrected for background binding of rAbs to blocking buffer only and data normalized and plotted as described above.

To assess the effect of glycolipids on MS rAb binding, green and red channel intensities were measured on all individual cells positive for intracellular PLP1 staining within an imaged field and binned according to costaining with O1 or O4 mAbs detected by Cy5 fluorescence. Background binding for each channel was subtracted using either the average binding of secondary control Abs in the absence of primary Abs or from G and R intensities obtained in areas of negative PLP1 intracellular staining. Because the nonspecific IC rAb IC#2 demonstrated no binding to live PLP1-transfected cells over a range of concentrations, we operationally defined positive binding of MS rAbs to PLP1^+^ cells in this set of experiments as any cell with a G/R ratio greater than 5 SDs above the mean G/R ratio (an average of 10 or more PLP1^+^ cells) obtained in the absence of primary MS rAbs. Statistical comparisons were performed using 2-tailed Student’s *t* test with Welch’s correction if necessary for unequal variances. The effect of cholesterol manipulation on MS rAb binding to PLP1-transfected cells was determined from images obtained from 3 or 4 independent experiments. Binding was normalized to G/R ratios obtained for vehicle only (RPMI), and data were statistically analyzed using 1-way ANOVA for unequal variances and Dunnett’s or Tukey’s correction for multiple comparisons.

A semiquantitative live-cell binding assay was used to measure CSF and peripheral blood IgG binding to PLP1-transfected HEKPE7 cells. Live-cell DAPI staining was used to identify and eliminate dead cells from the following analyses. Captured images were processed uniformly, and mean green and red channel intensities were determined from more than 8 single or small clusters of PLP1^+^ cells using ImageJ. To correct for CSF and PLP1 AA3 mAb binding to nontransfected HEKPE7 cells, mean green and red channel intensities were also measured on single or small clusters of PLP1^–^ cells within the same panel of images and subtracted to yield a corrected average G/R ratio. Each set of experiments always included a control binding of ON34 (40 µg/ml) that was used to normalize variations in green and red fluorescence intensities obtained between different experiments. To adjust values on a per-experiment basis, the mean ON#34 rAb-binding ratio obtained in experiment 1 was used as the standard value and divided by the ON34 ratio obtained in each subsequent experiment to obtain a correction factor that was multiplied by the average corrected G/R ratio to yield the normalized CSF- or CSF IgG–binding ratio (Supplemental Figure 8). Patient CSF considered positive for binding to PLP1-expressing cells required a normalized G/R ratio more than 3 SDs above the mean of inflammatory control CSF. Because there were no differences in the mean binding of inflammatory and noninflammatory control CSF or CSF IgG to PLP1-transfected cells, Fisher’s exact test was used to statistically compare the frequency of positive binding assays between the MS and control.

### Study approval.

CSF and serum were obtained from human patients with informed consent following a Colorado Multiple Institutional Review Board–approved (COMIRB-approved) protocol. Our protocol for the care, experimental treatment, and euthanasia of animals was approved by the University of Colorado Denver, Anschutz Medical Campus Institutional Animal Care and Use Committee (IACUC), which concurs with the NIH Guidelines for the care and use of Laboratory Animals (National Academies Press, 2011).

### Data availability.

Underlying experimental data can be accessed from the corresponding authors upon request. Values for all data points in graphs are reported in the Supporting Data Values file.

## Author contributions

GPO and JLB designed and coordinated the study and analyzed data. GPO organized and assisted in the execution of experiments and drafted the manuscript. TJF, AM, and VS contributed to in vivo microinjections, immunohistochemistry, and image analysis. KSG contributed to antigen identification and performed immunostaining of cerebellar slice cultures. KSG, KLS, AN, and KB contributed to animal breeding, in vivo microinjections, and image analysis. AMR, AM, and VS produced and purified MS rAbs. VS developed and perfected the myelin ELISA. GPO, WBM, EGH, and JLB reviewed data, provided experimental advice, and contributed to manuscript editing and revision. All authors read and approved the manuscript.

## Supplementary Material

Supplemental data

Supporting data values

## Figures and Tables

**Table 1:**
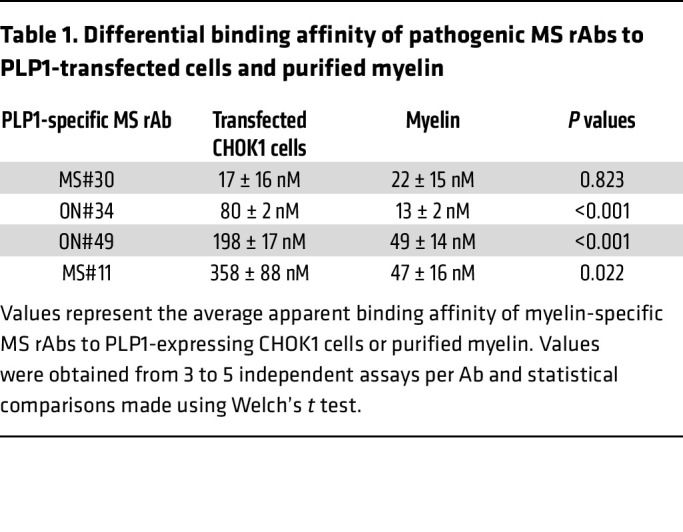
Differential binding affinity of pathogenic MS rAbs to PLP1-transfected cells and purified myelin

## Version 1 08/10/2023

In-Press Preview

## Version 2 10/02/2023

Electronic publication
