# Supplementary material for: Pathogenic myelin-specific antibodies in multiple sclerosis target conformational proteolipid protein 1–anchored membrane domains
Source: J Clin Invest. 2023 Oct 2;133(19):e162731. doi: 10.1172/JCI162731 (PMC10541191; doi:10.1172/JCI162731)
Supplement: Supplemental data [file jci-133-162731-s044.pdf]

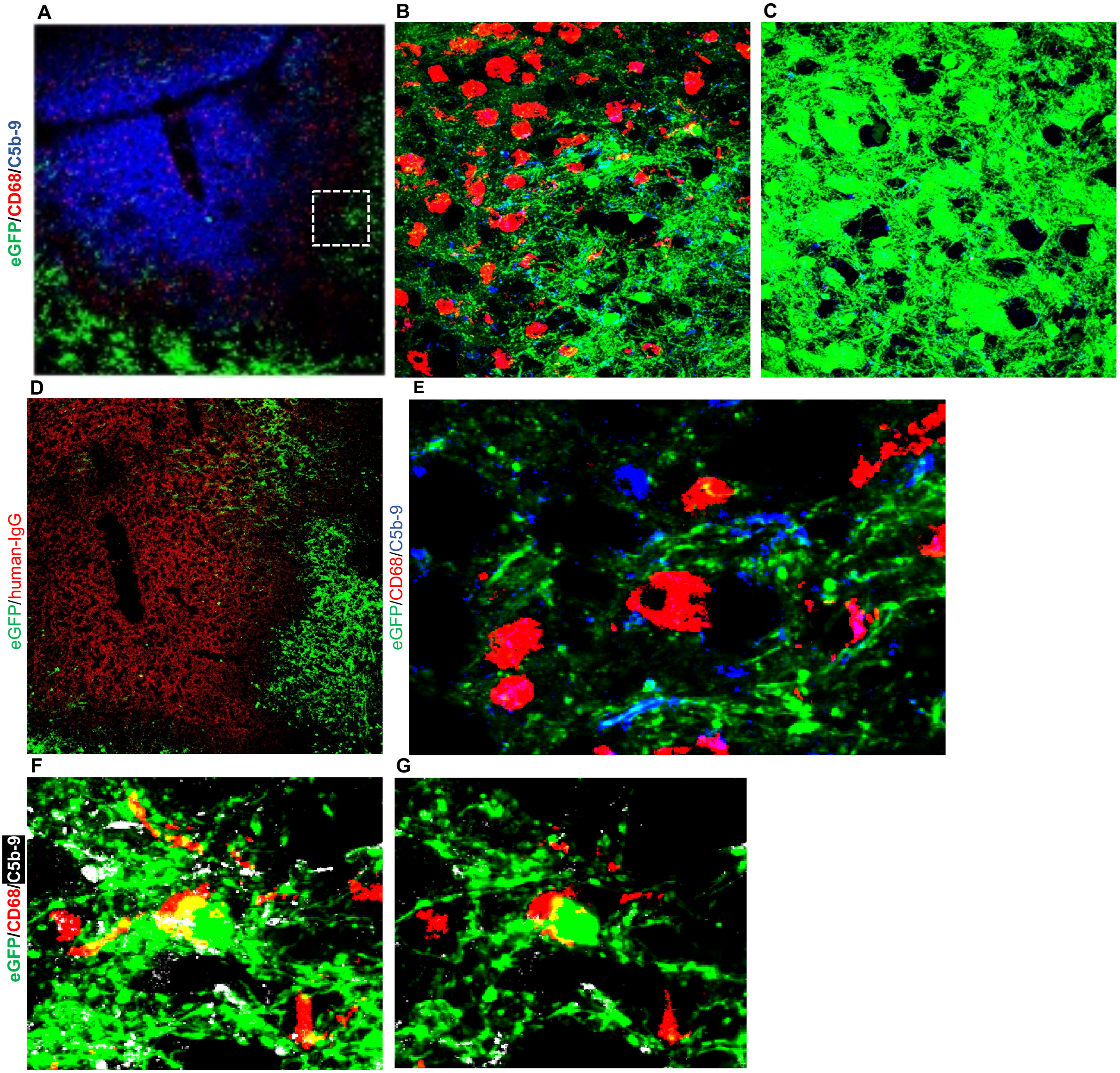

Figure S1. Phagocytes infiltrating demyelinating ON#34 lesion at 72 hours post-injection. (**A**) Image (10x) of terminal C5b-9 complement deposition (blue) within ON#34 lesion at 72h post-injection. **(B)** Confocal image (40x) of CD68 cell infiltration (red) and C5b-9 deposition near ON#34 lesion edge (boxed area) **(C)** Image of control hemisphere (CH) showing normal myelin and oligodendrocytes (green), absence of $CD68^{+}$ cells and C5b-9 staining; **(D)** Confocal image (10x) of recombinant IgG deposition (red) in area of oligodendrocyte cell loss; **(E)** Single plane confocal image (60x) of C5b-9 deposition (blue) on $eGFP^{+}$ myelin processes (green) and within adjacent $CD68^{+}$ phagocytes (red); **(F & G)** Consecutive single plane images of a $CD68^{+}$ phagocytic cell engulfing an $eGFP^{+}$ oligodendrocyte cell body in proximity to C5b-9 deposition (false colored in white).

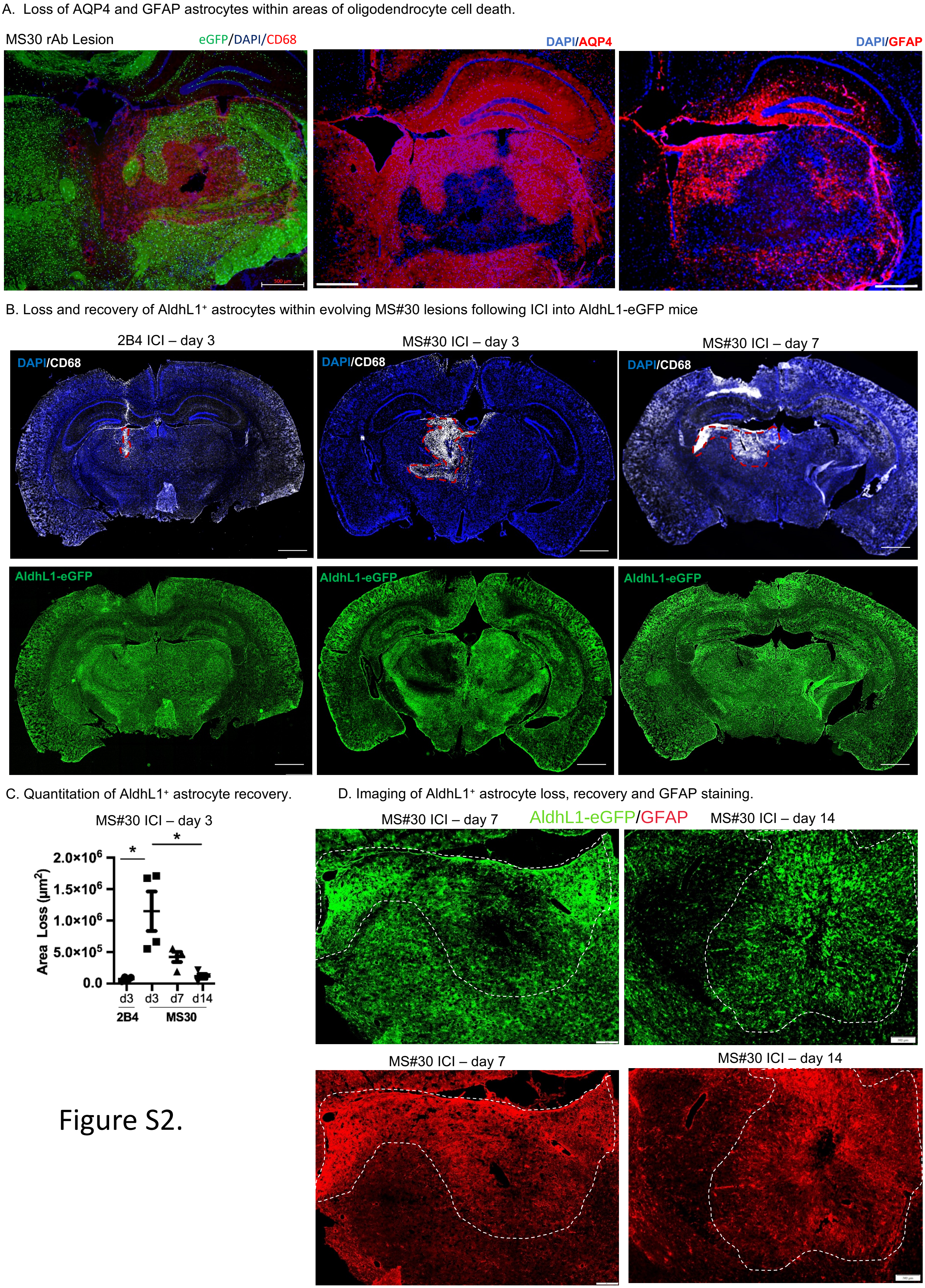
A. Loss of AQP4 and GFAP astrocytes within areas of oligodendrocyte cell death.
MS30 rAb Lesion
eGFP/DAPI/CD68
DAPI/AQP4
DAPI/GFAP
B. Loss and recovery of AldhL1⁺ astrocytes within evolving MS#30 lesions following ICI into AldhL1-eGFP mice
2B4 ICI – day 3
MS#30 ICI – day 3
MS#30 ICI – day 7
DAPI/CD68
DAPI/CD68
DAPI/CD68
AldhL1-eGFP
AldhL1-eGFP
AldhL1-eGFP
C. Quantitation of AldhL1⁺ astrocyte recovery.
MS#30 ICI – day 3
Area Loss (µm²)
2.0×10⁶
1.5×10⁶
1.0×10⁶
5.0×10⁵
0.0
*
*
d3
d3
d7
d14
2B4
MS30
D. Imaging of AldhL1⁺ astrocyte loss, recovery and GFAP staining.
MS#30 ICI – day 7
AldhL1-eGFP/GFAP
MS#30 ICI – day 14
MS#30 ICI – day 7
MS#30 ICI – day 14

Figure S2.

Figure S2. Transient astrocyte loss occurs in MS rAb + HC demyelinating lesions. (A) Images show loss of AQP4 immunostaining and absence of GFAP immunostaining in areas of eGFP oligodendrocyte loss in MS#30 + HC lesion. Bar = 500 μm. **(B)** Images of 2B4 and MS#30 + HC ICIs into Aldhl1 transgenic Swiss Webster mice at different time points post-injection. Infiltration of $CD68^{+}$ cells highlights the lesion boundaries and reveals differences in lesion severity between 2B4 and MS#30 ICIs. eGFP immunofluorescence identifies astrocyte cell bodies. Bar = 1 μm. **(C)** Graph shows significant loss of eGFP astrocytes between 2B4 and MS#30 lesions on day 3 post-injection (Mann-Whitney test). Significance of astrocyte recovery from day 3 to day 14 in MS#30 + HC ICIs was determined using one-way ANOVA. (D) Images of eGFP astrocyte recovery and GFAP expression in recovering day 7 and day 14 MS#30 ICIs. Dashed white lines identify corresponding areas of CD68 cell infiltraton. Bar = 200 μm.

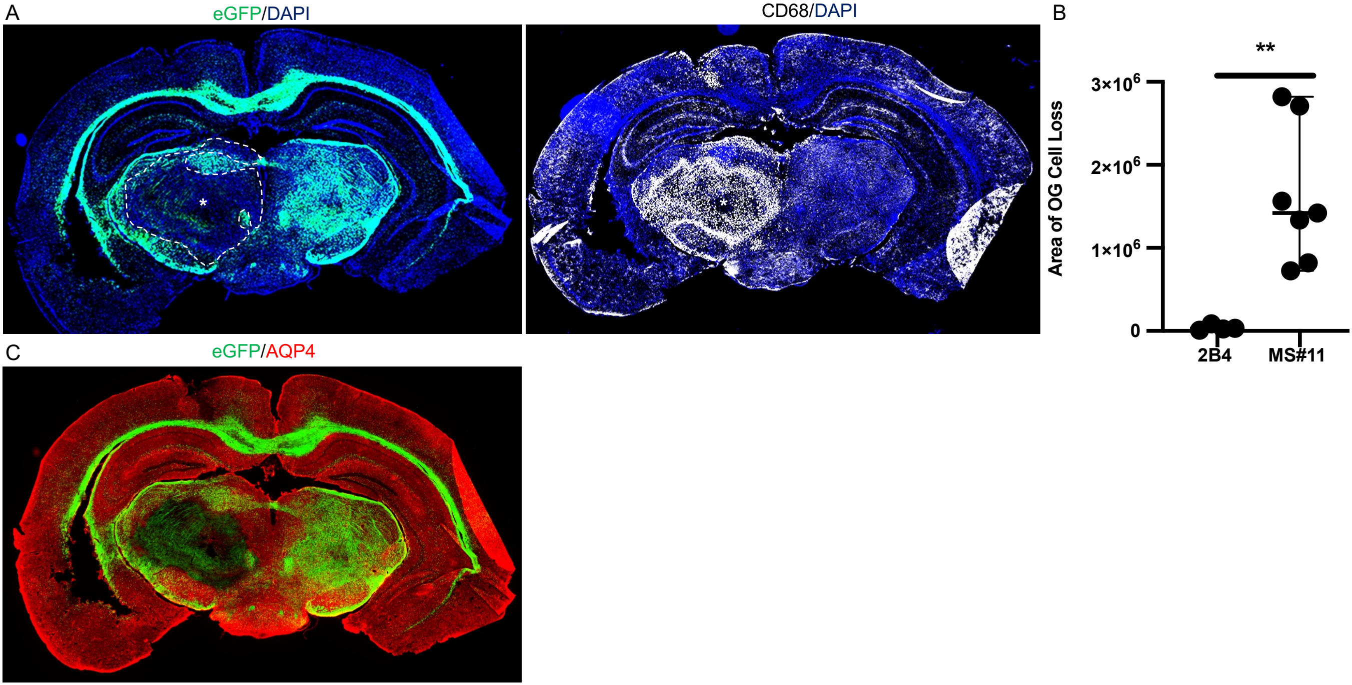

Figure S3. MS#11 is a pathogenic antibody to PLP1-transfected cells. (A) Injection of 5 ug MS#11 rAb with 20% HC produces large areas of oligodendrocyte loss and $CD68^{+}$ cell infiltration. **(B)** Graph compares area of oligodendrocyte loss between isotype control 2B4 and MS#11 ICIs (Mann-Whitney, **$p<0.01$) from a separate cohort of animals. Asterisk identifies injection site and hashed line outlines area of oligodendrocyte cell loss. **(C)** Image shows loss of AQP4 immunostaining in areas of eGFP+ oligodendrocyte cell loss.

Figure S3

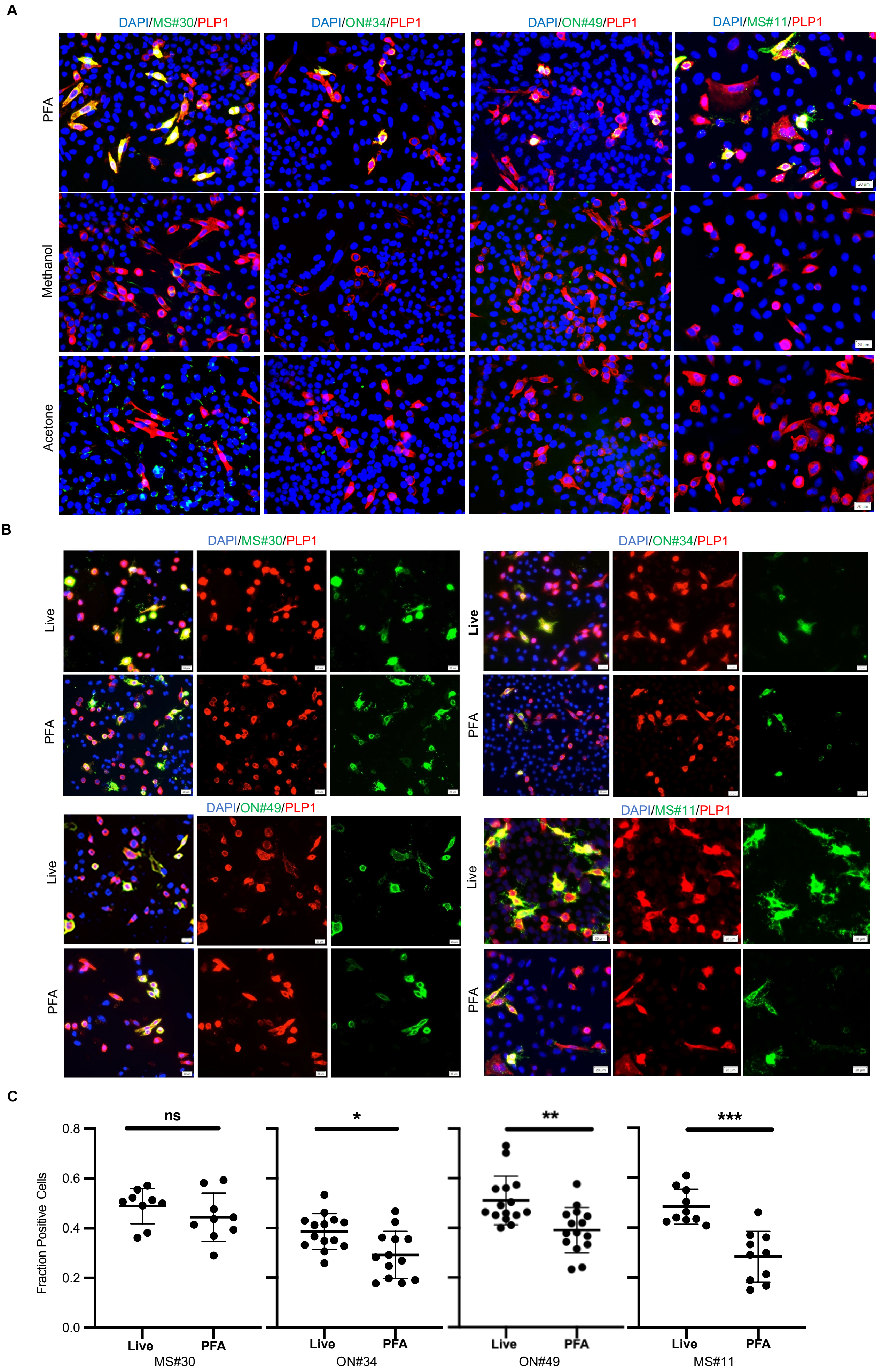
A
DAPI/MS#30/PLP1
DAPI/ON#34/PLP1
DAPI/ON#49/PLP1
DAPI/MS#11/PLP1
PFA
Methanol
Acetone
B
DAPI/MS#30/PLP1
DAPI/ON#34/PLP1
DAPI/ON#49/PLP1
DAPI/MS#11/PLP1
Live
PFA
C
Fraction Positive Cells
0.8
0.6
0.4
0.2
0.0
ns
*
**
***
Live
PFA
MS#30
ON#34
ON#49
MS#11

Figure S4. Conformational PLP1 epitopes are fixation sensitive and acquired post-translationally. (A) Immunostaining of PLP1-transfected cells with MS rAbs following fixation with paraformaldehyde, ice-cold methanol, or ice-cold acetone. **(B)** Images compare MS#30, ON#49, ON#34 and MS#11 rAb staining of live (upper panel) and PFA-fixed (lower panel) PLP1-transfected CHOK1 cells at 24 hr. post-transfection. **(C)** Graphs quantify the fraction of PLP1-transfected cells (mAb $AA3^+$ cells in red) co-stained with the indicated MS rAb (green). Data represents rAb (green) and PLP1 (red) cell counting of images from three independent transfection experiments. Comparison of means was performed using Student's t-test with Bonferroni correction for multiple comparisons.

PLP1 mAb MS#30 rAb MBP mAb

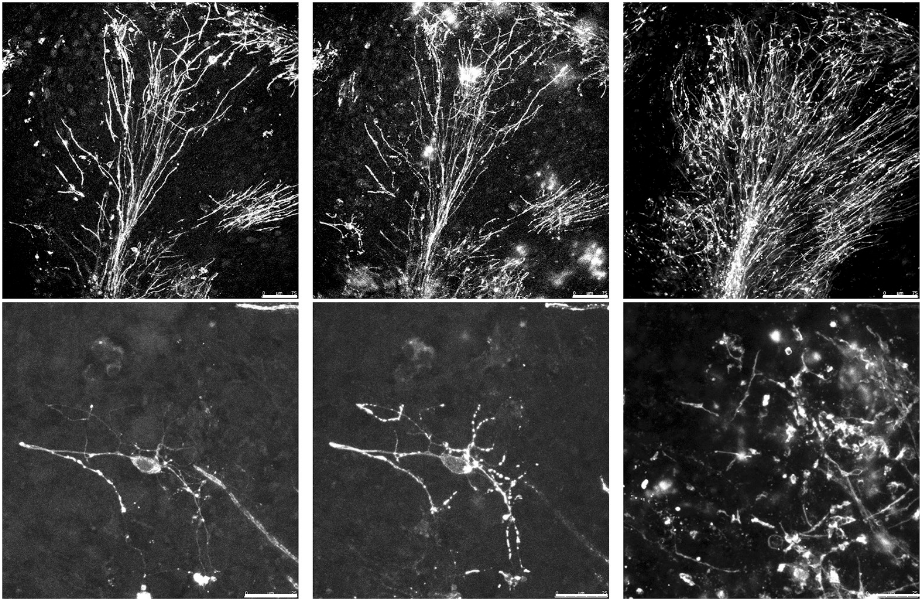

Figure S5. MS#30 immunostaining co-localizes with PLP1-expression in mosaic female PLP$^{x/-}$ C57Bl/6 mice. Confocal images (Bar = 100 μm) show immunostaining of folia from PFA-fixed cerebellar slice culture with PLP1AA3 mAb, MS#30 rAb and anti-MBP mAb. Because the PLP1 gene is located on the X chromosome, PLP1 expression in oligodendrocytes is mosaic. Staining of white matter tracts with MS#30 rAb coincides with the pattern of PLP1 expression whereas MBP staining can be seen throughout myelin processes.

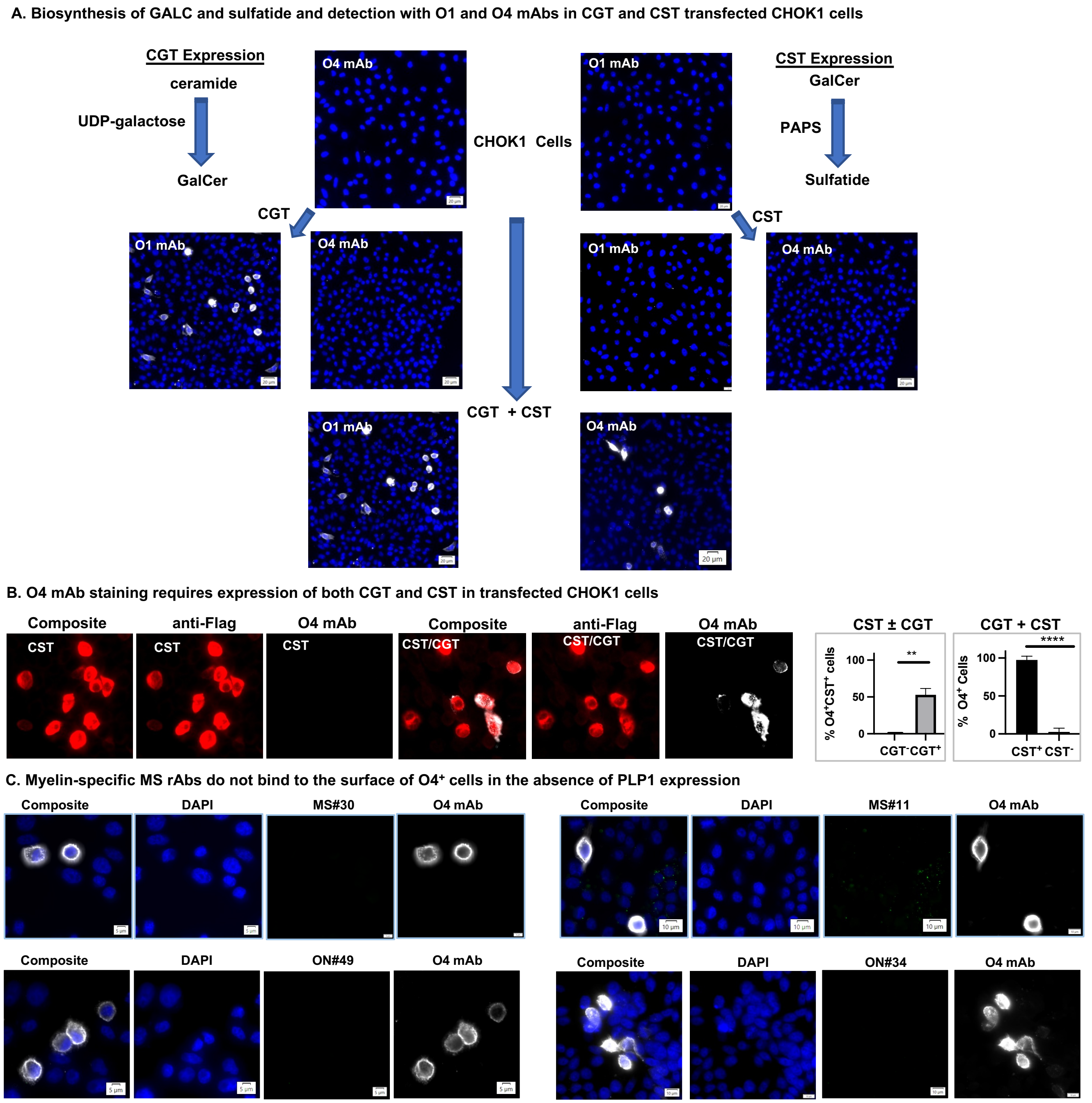

Figure S6. Oligodendrocyte developmental O1 and O4 mAbs identify cells expressing galactocerebroside and sulfatide respectively. (A) Flowchart and images of O1 and O4 mAb staining of live CHOK1 cells prior and following the individual and combined transfection of plasmid cDNA expressing galc and sulfatide biosynthetic enzymes CGT and CST. **(B)** Identification of CST-transfected cells using antibodies to a FLAG epitope (red) engineered onto the C-terminus of the CST expression construct. Graphs demonstrate that positive O4 staining of CHOK1cells requires expression of both CGT and CST enzymes and that O4 staining of CST + CGT transfections segregates with CST expression. Student's t-test,**p<0.01; ****p<0.0001. **(C)** Images show the absence of myelin-specific rAb binding to live CHOK1 cells following transfection with CGT and CST.

Figure S7. Effects of sulfatide and cholesterol on antibody binding to PLP1-expressing HEK293 cells.

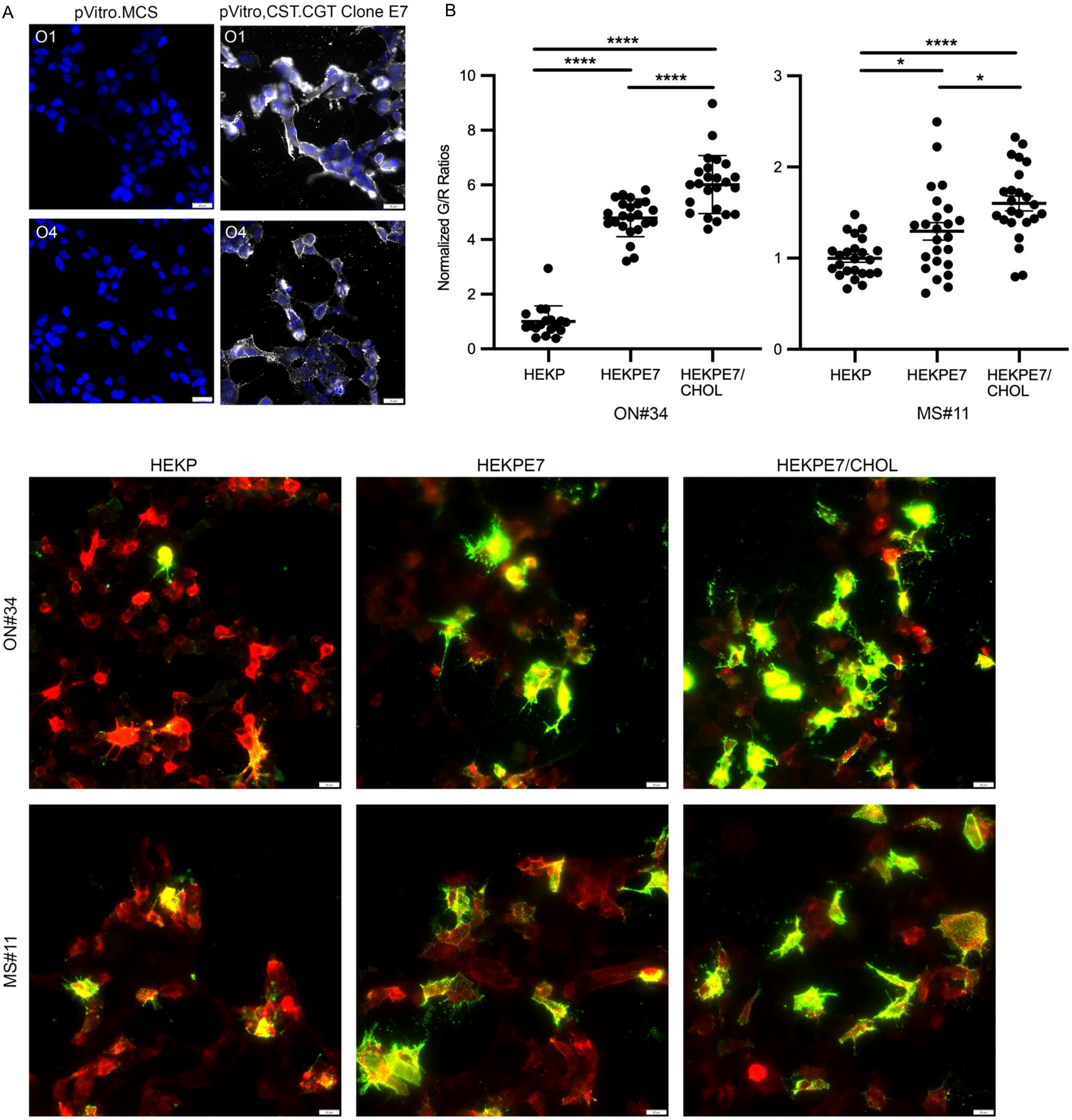

Figure S7. (A) Images of O1 and O4 mAb staining of stable cell lines derived from HEK293 parental cells (HEKP) containing naked dual expression pVitro-MCS plasmid DNA or with inserted CGT and CST cDNAs (clone E7). Bar = 20 µm. **(B)** Graphs and representative images show quantitative binding of ON#34 (40 µg/ml) and MS#11 (100 µg/ml) rAbs to AA3+ PLP1-transfected parental HEK293 cells (HEKP), clone HEKPE7 cells, and clone HEKPE7 treated with cholesterol. All G/R ratios were normalized to rAb binding to HEKP cells. Bar = 20 µm. Data represents replicate images obtained from two or three independent transfections statistically analyzed by one way ANOVA with Tukey's correction for multiple comparisons.

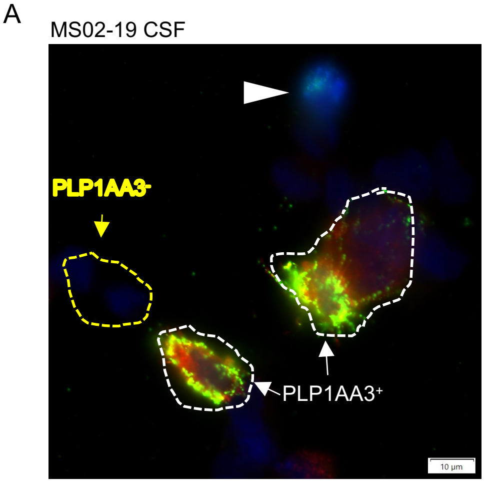

$$\text{Normalized G/R Ratio} = \frac{GF^{PLP1+} - GF^{PLP1-}}{RF^{PLP1+} - RF^{PLP1-}} \times \frac{\text{ON34 rAb G/R}^{Exp\ 1}}{\text{ON34 rAb G/R}^{Exp\ x}}$$

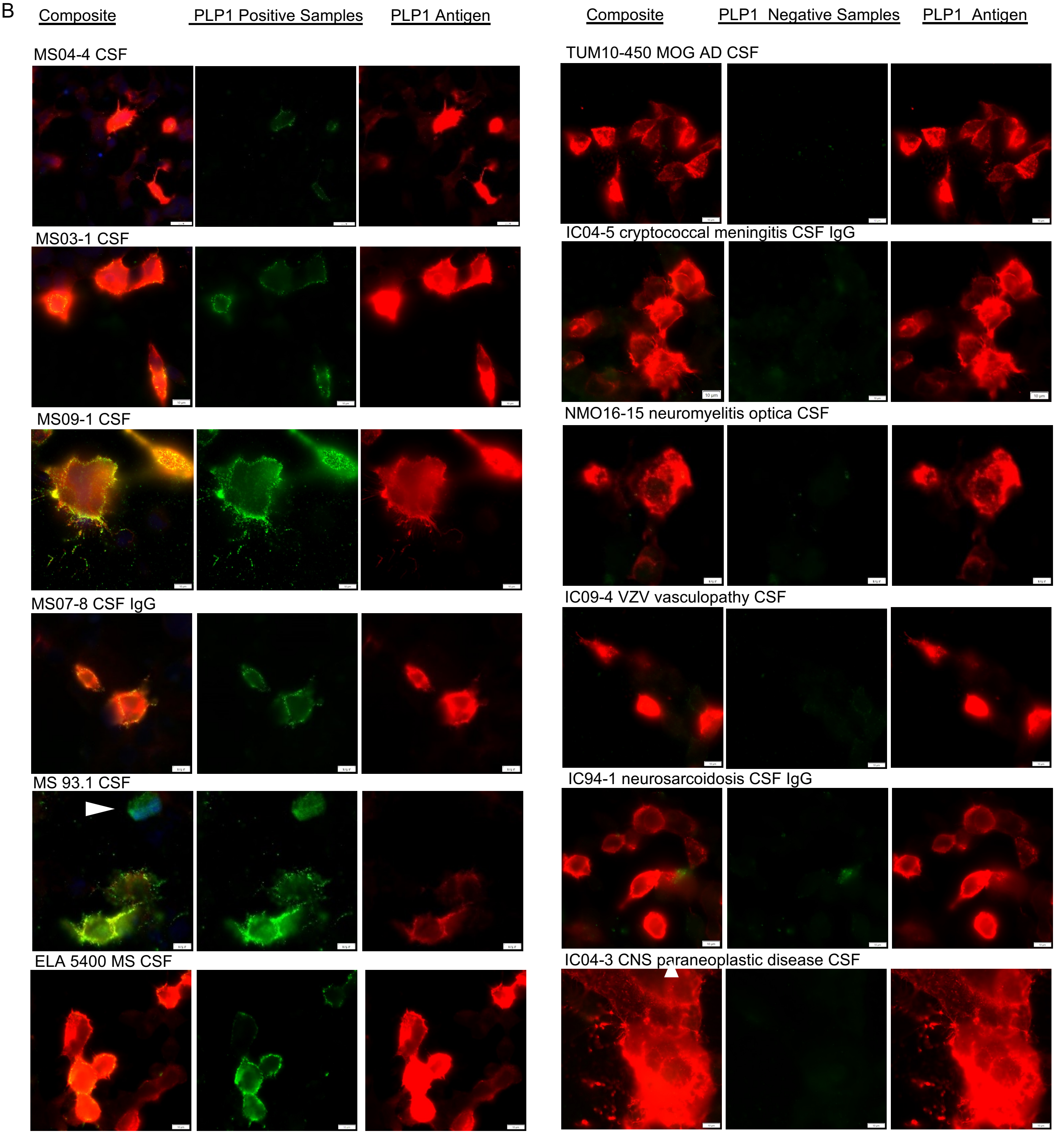

Figure S8. (A) Representative image of PLP1-transfected HEKE7 cells stained with MS02-19 CSF. The highlighted elements were used to calculate the normalized CSF G/R binding ratio. Bar = 10 µm. White dashed areas represent CSF IgG staining (green channel) of mAb AA3 positive (red channel) PLP1-transfected cells (white arrows). The mean green (GF) and red fluorescent (RF) staining intensities are measured from 8 or more $PLP1^+$ cells or clusters of cells per CSF sample and each corrected for background binding to AA3 negative cells, which are highlighted in this image by the yellow dashed line and arrow. Although not readily visible in this image, live non-transfected cells are identified from parallel images in which very dim DAPI staining can be enhanced. The white arrowhead identifies a bright DAPI positive dead cell, which is omitted from all analyses. To correct for inter-experimental variability in green and red channel staining intensities, each binding assay includes a positive control staining with ON34 rAb (40 µg/ml) at Bmax. The measured G/R ratio of ON34 binding obtained for our initial experiment (ON34 $rAb^{Day\ 1}$) is then divided by the binding ratio obtained for subsequent experiments (ON34 $rAb^{Day\ x}$) and used to compute a final normalized CSF G/R ratio as indicated in the accompanying equation. (**B)** Representative images of individual MS and control patient CSF or purified CSF IgG staining of PLP1-transfected HEKE7 cells. Bar = 10 µm. Images of MS04-4 and MS07-8 CSF staining are representative of weakly positive CSF and images of MS09-1 and MS93.1 represent more moderate to strong CSF positivity.

Table S1. Clinical, MRI and CSF Features of MS Patients used for CSF monoclonal recombinant antibody production[a]

Patient	Age	Sex	Diagnosis	White Matter Lesions	CSF Cells/µl	IgG Index	OCBs	PLP1^{+} rAbs[b]	Disease Duration at Lumbar Puncture	Time of Lumbar Puncture from Onset of Last Exacerbation
MS03-1[c]	25	F	Relapsing-remitting MS	5	0	1.4	6		6 months	2 months
MS04-2	26	F	Relapsing-progressive MS	36	0	1.7	5	MS#30<br>MS#11	6 months	1 month
MS05-3	30	F	Relapsing-remitting MS	4	18	1.7	21		14 months	2 months
ON07-7[c]	18	F	Relapsing-remitting MS	11	26	1.33	16	ON#34<br>ON#49	2 months	2 months
MS02-19	49	F	Primary-progressive MS	multiple	10	1.2	6		3 years	Not applicable

[a] Patients were not receiving immunomodulatory therapy at the time of lumbar puncture.

[b] Patient-derived rAbs from CSF plasmablast clones demonstrated to bind myelin and/or PLP1-transfected cells.

[c] CSF was collected and analyzed following their first clinical event. Patients subsequently developed relapsing-remitting MS within a 6-18 month period.

Table S2. Patient CSF Demographics			
**Patient Cohort**[a]	**Female**	**Male**	**Age ± SD**
**Multiple Sclerosis (**n =79)<br>RRMS (n = 59)<br>PPMS (n = 7)<br>SPMS (n = 5)<br>CIS (n = 2)<br>not classified (n = 6)	62	15	43 ± 11.6
**Neurologic Controls** (n = 81)<br>inflammatory (n = 45)<br>non-inflammatory (n = 39)	46	31	46.1 ± 12.5

[a]gender identity was unknown for two MS and four control patients.

Table S3. Screening of MS and inflammatory control CSF-derived rAbs for binding to PLP1-transfected cells

Patient	Diagnosis	CSF PLP1 G/R Ratio[a]	rAbs Assayed[b] CHOK1	HEKPE7	PLP1⁺
MS03-1	CIS/RRMS	0.221	15	4	0
MS04-2	RPMS	0.569	9	4	2
ON07-7	CIS/RRMS	0.714	6	6	2
MS05-3	RRMS	0.102	10	0	0
MS02-19	PPMS	1.345	9	8	0
Inflammatory Controls	SSPE,	0.007	3	1	0
	NMO	0.0262	7	0	0
	Meningitis	0.0062	2	1	0
	Pediatric OMS	ND	4	0	0
	VZV vasculopathy	ND	2	0	0

[a] Normalized G/R binding ratios of patient CSF to PLP1-transfected HEKPE7 cells are described in Fig. 9. NMO and SSPE rAbs were derived from multiple donors. G/R ratios are reported from only one of the CSF donors.

[b] Assays represent sampling of overlapping panels of rAbs (25 – 50 μg/ml) to PLP1-transfected CHOK1 cells or PLP1-transfected HEKPE7 cells supplemented with cholesterol. Positive staining was identified by visual and specific immunofluorescent rAb staining of $PLP1^+$ cells as shown in Fig. 3.

ND: assay was not performed
